# Carbohydrate Recognition Specificity of Trans-sialidase Lectin Domain from *Trypanosoma congolense*

**DOI:** 10.1371/journal.pntd.0004120

**Published:** 2015-10-16

**Authors:** Mario Waespy, Thaddeus T. Gbem, Leroy Elenschneider, André-Philippe Jeck, Christopher J. Day, Lauren Hartley-Tassell, Nicolai Bovin, Joe Tiralongo, Thomas Haselhorst, Sørge Kelm

**Affiliations:** 1 Centre for Biomolecular Interactions Bremen, Faculty for Biology and Chemistry, University Bremen, Bremen, Germany; 2 Africa Centre of Excellence for Neglected Tropical Diseases and Forensic Biotechnology, Ahmadu Bello University, Zaria, Nigeria; 3 Institute for Glycomics, Griffith University Gold Coast Campus, Queensland, Australia; 4 Shemyakin Institute of Bioorganic Chemistry, Russian Academy of Sciences, Moscow, Russia; Liverpool School of Tropical Medicine, UNITED KINGDOM

## Abstract

Fourteen different active *Trypanosoma congolense* trans-sialidases (TconTS), 11 variants of TconTS1 besides TconTS2, TconTS3 and TconTS4, have been described. Notably, the specific transfer and sialidase activities of these TconTS differ by orders of magnitude. Surprisingly, phylogenetic analysis of the catalytic domains (CD) grouped each of the highly active TconTS together with the less active enzymes. In contrast, when aligning lectin-like domains (LD), the highly active TconTS grouped together, leading to the hypothesis that the LD of TconTS modulates its enzymatic activity. So far, little is known about the function and ligand specificity of these LDs. To explore their carbohydrate-binding potential, glycan array analysis was performed on the LD of TconTS1, TconTS2, TconTS3 and TconTS4. In addition, Saturation Transfer Difference (STD) NMR experiments were done on TconTS2-LD for a more detailed analysis of its lectin activity. Several mannose-containing oligosaccharides, such as mannobiose, mannotriose and higher mannosylated glycans, as well as Gal, GalNAc and LacNAc containing oligosaccharides were confirmed as binding partners of TconTS1-LD and TconTS2-LD. Interestingly, terminal mannose residues are not acceptor substrates for TconTS activity. This indicates a different, yet unknown biological function for TconTS-LD, including specific interactions with oligomannose-containing glycans on glycoproteins and GPI anchors found on the surface of the parasite, including the TconTS itself. Experimental evidence for such a scenario is presented.

## Author Summary

In this study we demonstrated the binding of TconTS lectin domains (TconTS-LD) to high-mannose *N*-glycans and provide evidence for a biological function for this interaction. TconTS1 and TconTS2 lectin domain bind to galactosyl as well as mannosyl glycans with different affinities as shown by glycan array analysis. Along this line, we have also demonstrated binding of TconTS-LD to high-mannose *N*-glycans on glycoproteins, which is competitively inhibited by the corresponding free high-mannose *N*-glycans, underlining the TconTS-LD substrate specificities. TconTS1 dimerisation is mediated by TconTS-LD binding to its *N*-glycans and enzymatic *N-*deglycosylation of TconTS leads to an increase of monomers. STD NMR results obtained indicate that oligo-mannosylated glycans bind to TconTS-LD to a different site than lactose. In summary, this is good evidence that the lectin domains of TconTS1 and TconTS2 play relevant roles modulating the biological functions of these and possibly other trans-sialidases. Further detailed analysis on TconTS-LD and its role in enzyme activity will lead to a better understanding and possibly to new strategies against Nagana in livestock.

## Introduction

The protozoan parasite *Trypanosoma congolense* is the most prevalent cause of animal African Trypanosomiasis (AAT), also called Nagana in cattle and other livestock, causing death to millions of animals resulting in huge economic losses [1–3]. During the parasite’s life cycle in the mammalian host and the tsetse fly vector *T*. *congolense* undergoes different developmental stages utilising various strategies to escape the defence systems of both host and vector. For instance, trypanosomes are unable to synthesise sialic acid (Sia) [4], instead *T*. *congolense*, like several other trypanosomatids, expresses an unusual glycosyl-transferase called trans-sialidase (TS) that transfers Sia from host cell glycoconjugates to its own surface structures [5,6].

TS are found in both the African and South American trypanosomes [7–10]. However, their roles in parasite development and pathogenesis appear to be species dependent, as the relevance of TS or sialidase activities has been shown for nagana caused by *T*. *congolense* [9], but not for sleeping sickness caused by *T*. *brucei ssp*. The TS from *Trypanosoma cruzi* (TcTS), the causative agent of Chagas' disease in humans [11], is the best characterised [12–18] with the mechanism of sialic acid transfer and catalytic activity being described in detail. It has been suggested that the catalytic domain (CD) of TconTS is located at the N-terminus and folds into a β-propeller structure, similar to that of known bacterial and viral sialidases [19–21]. The CD of trypanosomal TS is presumed to be linked via a well-conserved, relatively long α-helix (22 to 25 amino acids) to a C-terminal domain, whose function has remained unclear. The crystal structure of TcTS [14] revealed that the C-terminal domain folds into a β-barrel topology similar to that of known plant lectins such as GS4 (*Griffonia simplicifolia* lectin 4) [22], GNA (*Galantus nivalis agglutinin*, Snowdrop lectin) [23], LOL (*Lathyrus ochrus* lectin) [24] and WGA (*Wheat germ agglutinin*) [25]. This structural similarity suggests that the C-terminal domain may be a potential carbohydrate-binding site or “lectin-like” domain (LD). In contrast to TcTS, only a few studies have investigated the enzymatic activities of *T*. *brucei* TS (TbTS) [26–29] and TconTS [6,30,31]. However, given the overall high primary sequence similarity of all TS at the catalytic site it can be assumed that the molecular mechanisms of the African TS are similar to those described for TcTS.

In addition to several other TS-like genes, *T*. *congolense* possesses eleven TS1 (TconTS1) genes encoding variants with 96.3% overall amino acid identity [30]. Recombinant TconTS1 variants are able to desialylate fetuin in the presence of lactose to generate α2,3-sialyllactose (3‘SL), demonstrating not only their TS activity but also their sialidase activity, reflecting their ability to hydrolyse terminal sialic acids [30]. Three additional *T*. *congolense* TS family members, TconTS2, TconTS3 and TconTS4, have recently been described, sharing more then 40% amino acid identity [31]. Furthermore, kinetic data show that all TconTS investigated so far have different affinities for glycoprotein and oligosaccharide substrates [30,31].

Previous research on trypanosomal TS has been focussed on investigating the TS CD with respect to substrate specificities, mechanisms of sialic acid transfer and sialidase activities [6,12,14,27,30–34]. However, apart from sequence and structural data, limited information regarding the LD of the trypanosomal TS is available, with the actual function of the LD in TS remaining unknown. Due to structural similarities with known lectins, it had been proposed [13,35] that TS LD binds carbohydrates and may play a role in mediating cell adhesion. Recently, Ammar et al. suggested that TconTS lectin like domain binds sialic acids and is involved in endothelial cell activation [36]. However, to the best of our knowledge no direct evidence for carbohydrate-binding specificities of TS LD has been described.

Interestingly, a detailed phylogenetic analysis comparing TS domains revealed that the TconTS-CDs grouped the highly active TconTS together with the less active enzymes. In contrast, when aligning TconTS-LDs, the highly active TconTS grouped together [31], indicating a potential role of TconTS-LD in modulating enzyme activities.

Here we report on the biochemical characterisation of four recombinant TconTS-LD (TconTS1-4) employing glycan array, STD NMR and binding/inhibition assays that identified TconTS-LD as a carbohydrate recognition domain (CRD), with several oligosaccharides identified as TconTS-LD binding partners. Interestingly, prominent ligands involved were oligosaccharides with terminal mannose, which are not substrates for trypanosomal TS activity, suggesting previously not reported biological functions. In addition, we provide strong evidence for a second binding site for oligosaccharides with terminal galactose moieties.

## Results

### Expression of recombinant TS-LD

**Fig 1:**
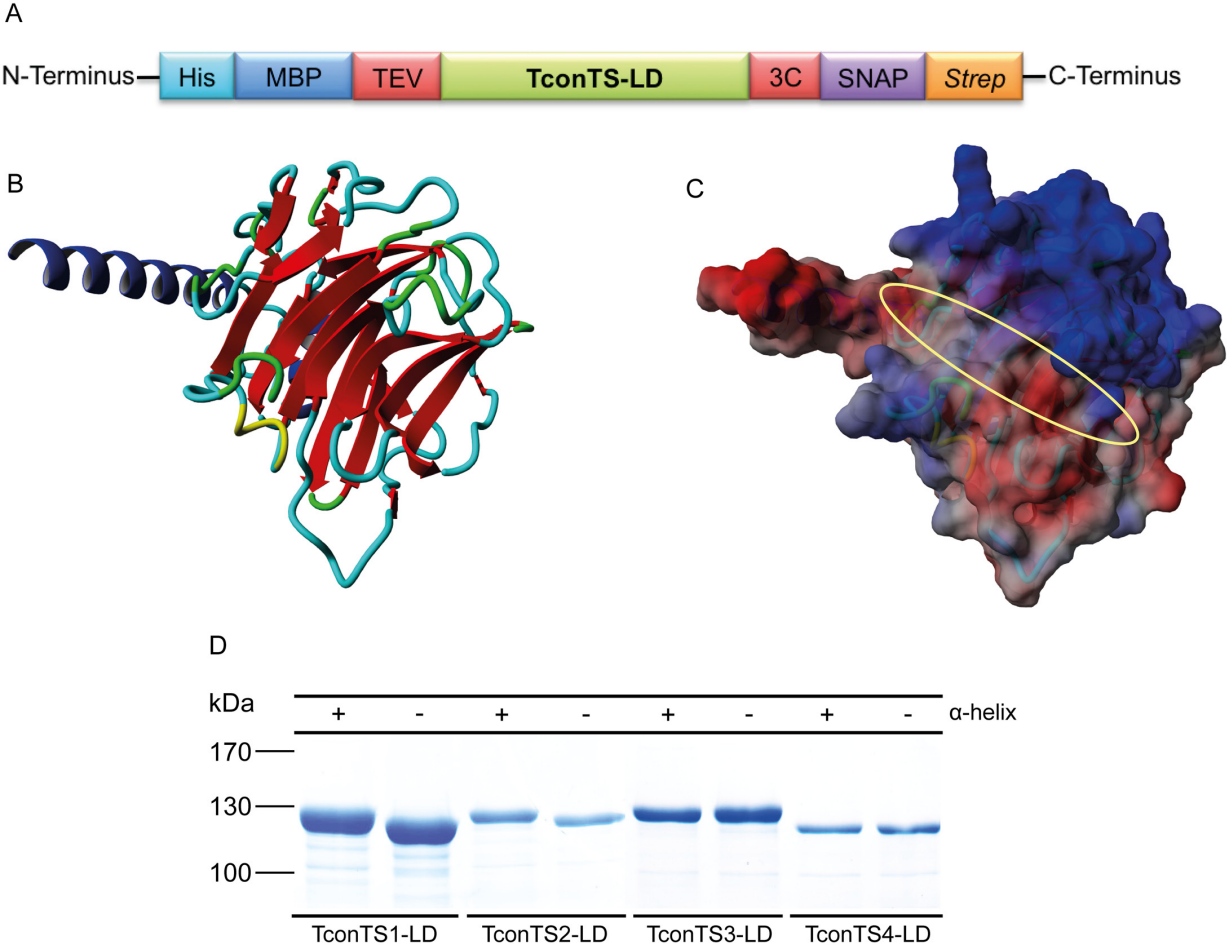
Generated TconTS-LD proteins. A: Schematic presentation of recombinant TconTS-LD fusion proteins expressed in bacteria. Fusion tags flanking TconTS-LD are: His: poly histidine tag, MBP: maltose binding protein tag, TEV: *tobacco etch virus* protease cleavage site, 3C: human rhinovirus 3C protease cleavage site, SNAP: SNAP-tag, *Strep*: *Strep-tag*. B: Homology model of TconTS2-LD comprising the α-helix calculated using TconTS2 amino acid sequence and crystal structure of *Trypanosoma cruzi* TS (PDB code: 3b69) as template employing the software Yasara. C: The molecular electrostatic surface of the homology model (B) was calculated using the ESPPME method of Yasara structure. Red colour indicates a positive potential, blue a negative and grey a neutral. A yellow ellipse indicates the groove encompassing the proposed binding site. D:SDS-PAGE of purified TconTS-LD proteins. After expression in *E*. *coli* Rosetta pLacI, 1–2 μg double affinity purified recombinant TconTS-LD, containing and lacking the α-helix, were loaded in each lane of an 10% SDS polyacrylamide gel as indicated. After electrophorese, the gel was stained using Coomassie Brilliant Blue.

To characterise the LDs of TconTS the gene sequences encoding the TconTS1-LD, TconTS2-LD, TconTS3-LD and TconTS4-LD were subcloned into a modified pET28a bacterial expression vector as described under Methods. All proteins comprise a N-terminal poly histidine tag (His-tag) directly attached to maltose binding protein (MBP) as well as C-terminal SNAP- and Strep-tags. In these proteins, the tags flanking TconTS-LD can be enzymatically cleaved using the tobacco etch virus (TEV) and human rhinovirus 3C (HRV 3C) proteases (Fig 1A).

Recombinant protein comprising only His-MBP-SNAP-Strep, but no TconTS-LD was used in control experiments. For TconTS-LD interaction studies, based on homology models (Fig 1B and 1C) two sets of constructs were generated with or without the α-helix connecting CD to the LD (Table 1), to investigate its potential influence on binding activity. Expression conditions were optimised for efficient production of soluble TconTS-LD in amounts ranging from 0.5–2 mg/L bacterial culture as described under Methods. After tandem affinity chromatography employing Ni-NTA and Strep-tag consecutively, protein purity was confirmed by gel electrophoresis and Western blot analysis (Fig 1D). All TconTS-LD were obtained as pure proteins clearly showing a migration shift due to the presence or absence of the N-terminal α-helix.

**Table 1:** Bacterial and eukaryotic expressed recombinant TconTS constructs.

		Expression system		Domains					
Nr.	TconTS	*E*. *coli*	CHO-Lec1	CD	αHel	LD	Length* (AA)	MW (kDa)	Abbreviation
1	1	+	-	-	+	+	258	27.5	TconTS1-αHel-LD
2	1	+	-	-	-	+	236	25.1	TconTS1-LD
3	2	+	-	-	+	+	263	29.3	TconTS2-αHel-LD
4	2	+	-	-	-	+	238	26.5	TconTS2-LD
5	3	+	-	-	+	+	250	27.2	TconTS3-αHel-LD
6	3	+	-	-	-	+	225	24.3	TconTS3-LD
7	4	+	-	-	+	+	261	28.1	TconTS4-αHel-LD
8	4	+	-	-	-	+	236	25.3	TconTS4-LD
9	1	-	+	+	+	+	711	77.5	TconTS1
10	2	-	+	+	+	+	694	77.0	TconTS2
11	3	-	+	+	+	+	682	74.7	TconTS3
12	4	-	+	+	+	+	747	82.7	TconTS4

* Length of the proteins are given in number of amino acids (AA) for the TconTS part excluding the N- and C-terminal tags.

### Screening and identification of glycans as potential TconTS-LD ligands

**Fig 2:**
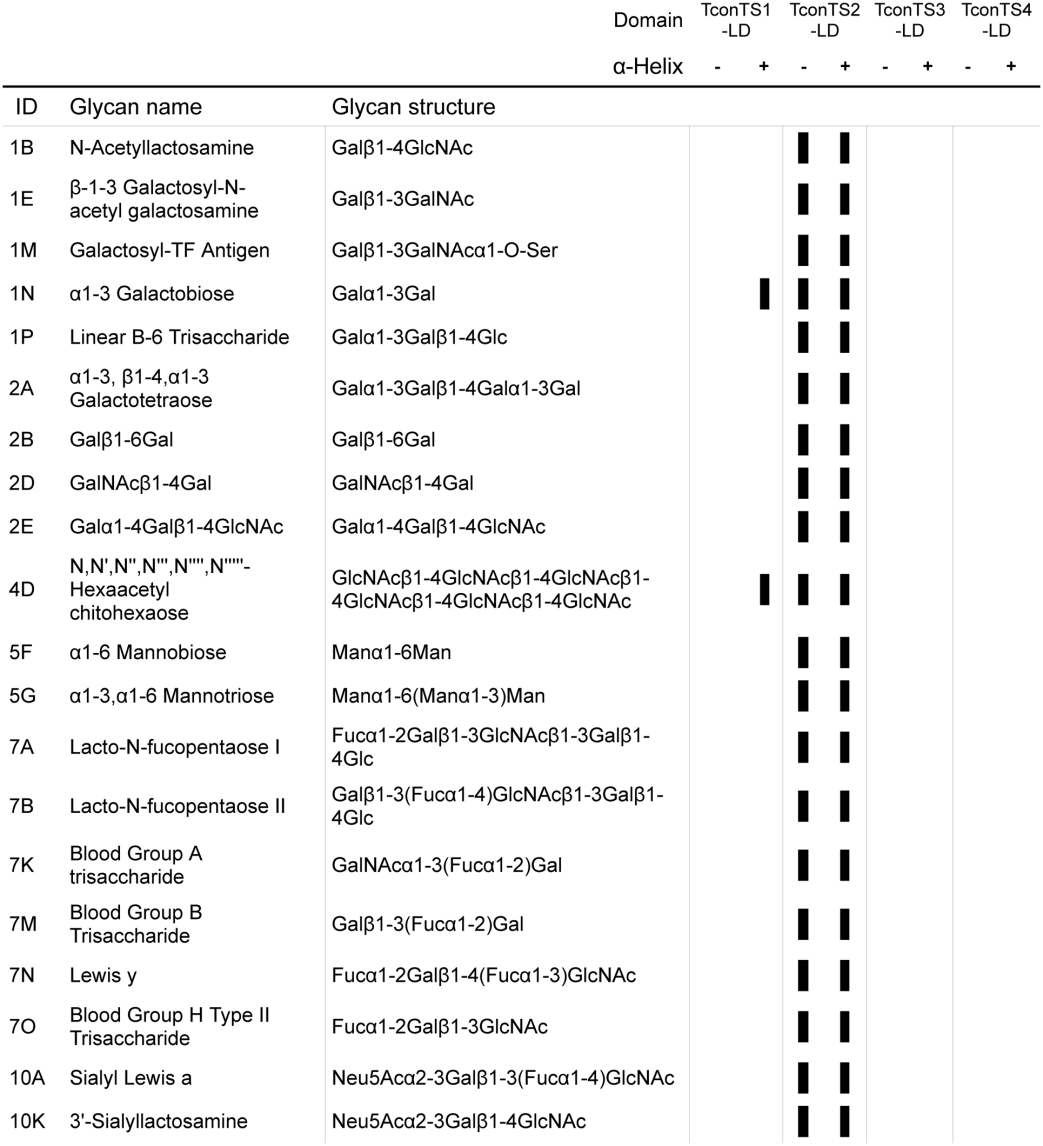
Summary of TconTS-LDs binding to glycans as determined by glycan array analysis. TconTS-LDs binding to the glycan arrays was determined as described under Methods. Black bars indicate glycans bound by the TconTS-LDs. The presence and absence of the α-helix in TconTS-LD constructs is indicated with “+” and “-“, respectively. Further binding data (S2 Fig) and all glycans on the arrays (S1 Table) are available as Supporting Information.

Glycan array analysis was performed to identify potential TconTS-LD oligosaccharides binding partners. Recombinant TconTS-LD containing His and MBP fusion tags (Table 1 and Fig 1A) were pre-complexed with anti His mouse polyclonal antibody, anti-mouse-IgG-TexasRed conjugated rabbit polyclonal antibody and anti-rabbit-IgG-TexasRed conjugated donkey polyclonal antibody. These were then applied to glycan arrays printed onto SuperEpoxy2 glass slides comprising 367 diverse biologically relevant glycan structures (S2 Fig). The major subset of glycans bound by TconTS-LD are summarised in Fig 2 (full binding data provided in S2 Fig and S1 Table). As expected, initial glycan array experiments revealed signals associated with maltose, maltotriose, isomaltotriose, maltotretraose, isomaltotetraose and related glycans due to the binding of MBP (S1A Fig). Therefore, 10 mM maltose was added as a competitor during binding and washing steps to inhibit the MBP interaction with maltose and related structures present on the arrays. Under these conditions the majority of maltose related signals disappeared. Only some signals for maltotriose, maltotetraose and other maltodextrins remained. Given that maltotriose has a more than 6-fold higher affinity for MBP (*K*_*d*_: 0.16 μM) compared to maltose (*K*_*d*_: 1 μM) [37], 10 mM maltotriose instead of maltose was used during binding and 1 mM in all wash steps. Under these conditions, binding of MBP to all remaining maltose related structures was successfully inhibited (S1B Fig). Another option that could have been used to prevent MBP associated binding to our glycan array would have been a proteolytic cleavage using the TEV protease cleavage site of the recombinant TconTS-LD protein (Fig 1A). However, the removal of the MBP-tag and subsequent purification of TconTS-LD leads to low yield of pure TconTS-LD, since often the protease digest is not complete. Therefore, we choose to inhibit MBP binding to maltose-related structures on the glycan arrays with maltotriose in the analyses of all eight TconTS-LD constructs. Glycan array analysis of TconTS2-αHel-LD and TconTS2-LD showed clear binding to several different galactobiose and lactose containing oligosaccharides, as well as to some of their *N-*actetylamine derivatives listed in Fig 2. Also several fucosylated, and two sialylated glycans were bound, although the binding to these structures was less pronounced compared to unsubstituted *N*-acetyllactosamine. Whereas binding to potential TS substrates containing galactose was not unexpected, surprisingly, we also observed binding to α1-6-mannobiose and α1–3,α1-6-mannotriose, which was similar for TconTS2-LD with and without the α-helix. No obvious preference of TconTS2-LD for any of the oligomannose isomers present on the array was identified. The number of glycan structures bound by TconTS1-LD was lower than that observed for TconTS2-LD, and no binding to any glycan structures was observed for either TconTS3-LD or TconTS4-LD under the conditions used.

### STD NMR studies provide evidence for a secondary binding site in TconTS-LD

**Fig 3:**
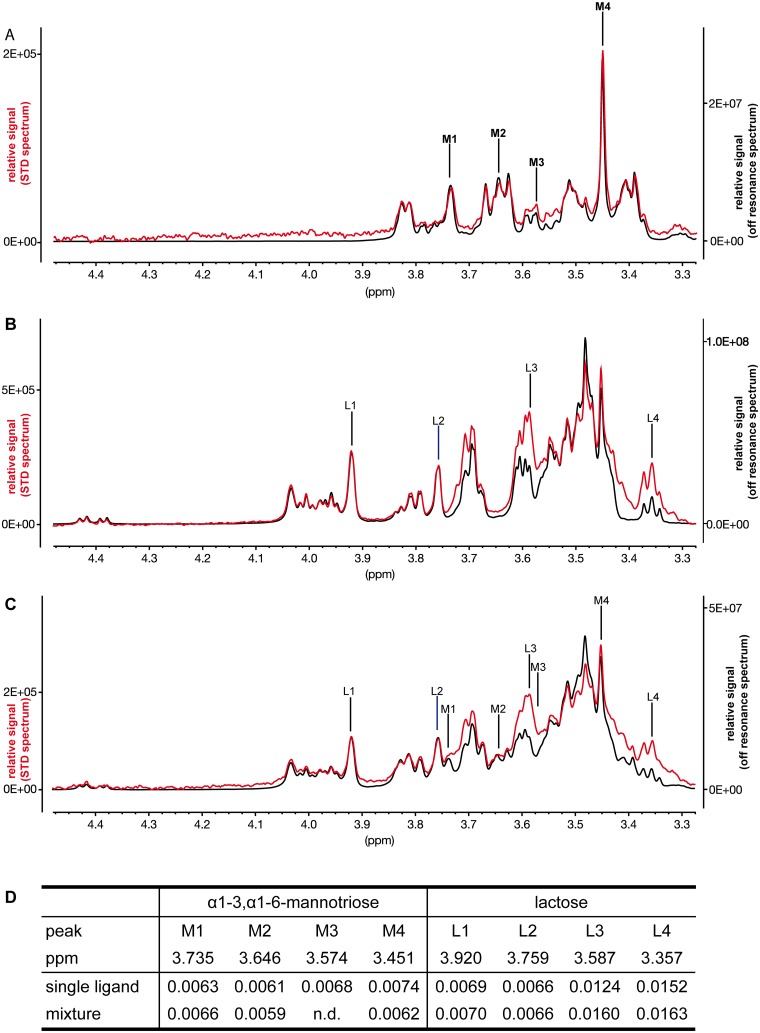
STD NMR experiments of TconTS2-LD. STD NMR experiments with 5.5 μM TconTS2-LD were performed as described under Methods. Off-resonance (black lines) and STD NMR (red lines) spectra are shown. A: In the presence of 1.73 mM for 3α,6α-mannotriose; B: In the presence of 3.45 mM lactose; C: In the presence of 1.73 mM 3α,6α-mannotriose and 1.73 mM lactose. D: STD NMR effects for the signals indicated were determined as ratios between the intensities at the indicated ppm in the off-resonance spectra and corresponding STD NMR spectra using the software TopSpin 3.2. M1, M2, M3 and M4 stand for the NMR signals of 3α,6α-mannotriose, and L1, L2, L3 and L4 for those of lactose, for which the STD NMR effects are shown either for the single ligands (spectra shown in A and B) or for the mixture (spectra shown in C). n.d.: not determined, since in the ligands mixture the STD effect for M3 could not be determined from the spectra.

TconTS2-LD showed the highest lectin activity on glycan arrays. Therefore, in further experiments we focused on TconTS2-LD to more fully characterise and confirm the binding of TconTS-LD to both galactose and mannose containing oligosaccharides observed on the glycan array. Several NMR-based methods have been employed to investigate protein carbohydrate interactions on a structural level. For example, line broadening and peak shifts of ^1^H-NMR signals from amino acid side chains provide information on the type of amino acids involved as well as the occupation of the binding site and thus equilibrium kinetic data, as has been shown for Siglec–1 [38]. Saturation transfer difference (STD) NMR experiments provide important information on the binding epitope of the complexed carbohydrate ligand, since the relative signal intensities of the difference spectra provide direct information on the proximity of the affected protons to the protein [39]. Protein signals are selectively saturated at -1.00 ppm (on-resonance) and subtracted from an off-resonance spectrum (30 ppm) resulting in the final STD NMR spectrum revealing only protons and functional groups of a binding ligand that are in close proximity to the protein surface. Therefore, STD NMR has been widely used to analyse the binding of lectins to their specific carbohydrate ligands. Lactose and α1–3,α1-6-mannotriose were used as ligands for TconTS2-LD as described under Methods. Fig 3A shows the ^1^H NMR (off resonance) and STD NMR spectra of α1–3,α1-6-mannotriose. The relative signal intensities of the STD spectrum (red line) are almost identical to those of the oligosaccharide ^1^H NMR spectrum (black line). Binding of lactose to TconTS2-LD was also clearly observed (Fig 3B). It is important to note that relatively strong STD NMR signals at 3.36 ppm (β-GlcH2) and at 3.92 ppm (GalH4) provide good evidence that both monosaccharide units of lactose are in close contact with the protein.

Taken together, the STD NMR data confirmed binding of both lactose and α1–3,α1-6-mannotriose to TconTS2-LD, which were initially identified by glycan array analysis, and raises the question as to whether both oligosaccharides bind to the same or distinct sites on TconTS2-LD. To try and address this question, an additional STD NMR competition experiment was performed, where an equal quantity of lactose was added to the TconTS2-LD/α1–3,α1-6-mannotriose complex. If lactose was able to bind to the same site as α1–3,α1-6-mannotriose, the two oligosaccharide ligands would compete, which then would lead to a reduction in the STD NMR signals for one or both ligands, depending on their relative affinities for this site [40]. However, no such reduction was observed for either lactose or α1–3,α1-6-mannotriose (Fig 3C and 3D) suggesting that both ligands are likely to bind simultaneously to different binding sites on TconTS2-LD.

### Binding of TconTS-LD to mannosylated glycoproteins

**Fig 4:**
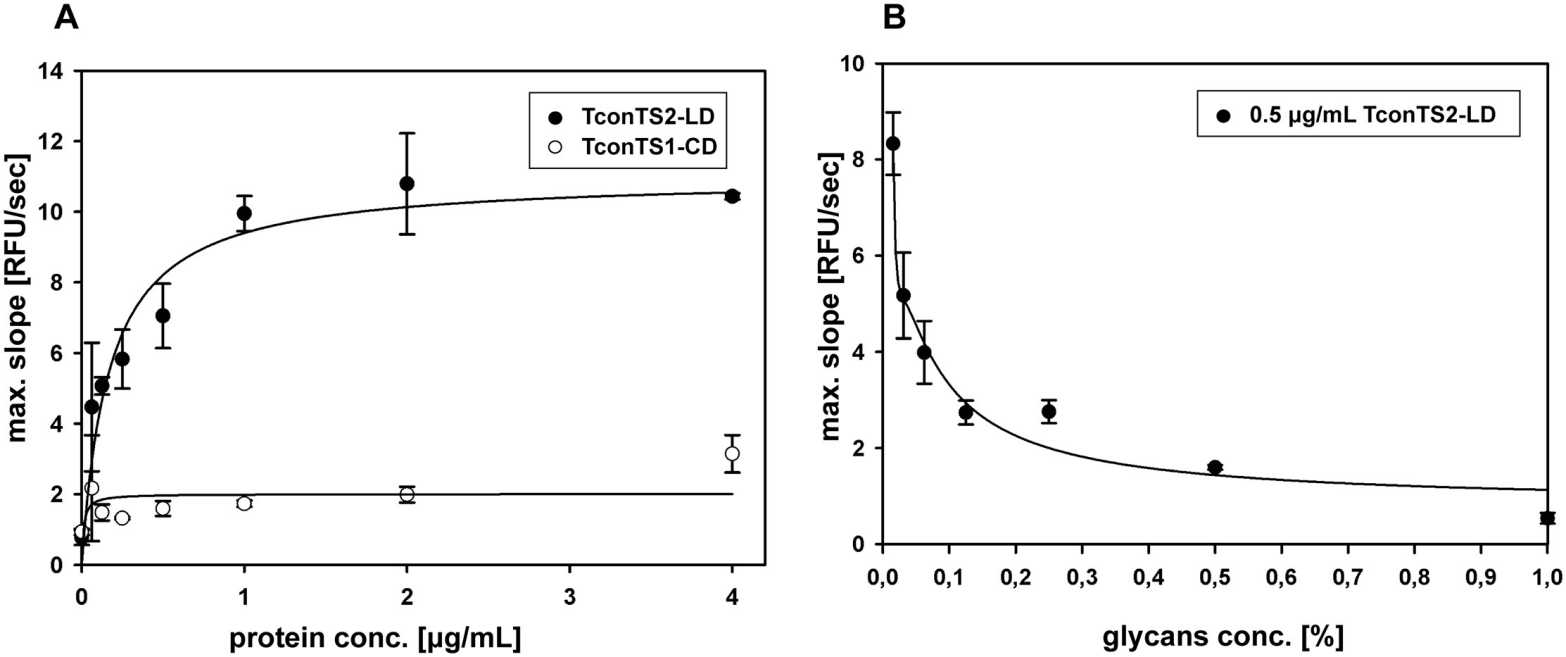
Binding specificity of TconTS2-LD. A: TconTS2-LD concentration dependent binding to immobilised huS2-Fc (5μg/mL). B: Competitive inhibition of TconTS2-LD binding to huS2-Fc in the presents of serially diluted high-mannose *N*-glycans. Undiluted inhibitor solution was set to 1.0. The maximum increase in relative fluorescence units (RFU) over time was determined as described under Methods. Data points are means ± standard deviation of triplicates.

We established microtitre plate based binding and inhibition assays to further characterise TconTS-LD binding affinity and specificity. Our glycan array and STD NMR experiments revealed TconTS-LD binding to oligo-mannose oligosaccharides. To further investigate how this specificity mediates interactions of TconTS-LD with glycoproteins, recombinant human Siglec 2 (huS2-Fc, described under Methods) expressed in Chinese hamster ovary Lec1 (CHO-Lec1) cells was used as a model glycoprotein. Due to the lack of *N*-acetylglucosaminyltransferase 1 (GnT1) CHO-Lec1 cells are unable to synthesise complex and hybrid *N-*glycan structures. Therefore, these proteins contain only high-mannose glycans of the type Man_5_GlcNAc_2_-Asn [41]. Purified huS2-Fc immobilised in microtitre plate wells was incubated with different concentrations of TconTS2-LD and binding was detected as described under Methods. TconTS1-catalytic domain (TconTS1-CD) was used as a control for binding specificity. As shown in Fig 4A, concentration-dependent binding of TconTS2-LD to immobilised huS2-Fc was clearly observed, reaching a maximum intensity at approximately 2 μg/mL TconTS2LD due to saturation of the binding sites. Wells without immobilised huS2-Fc were used as a control. No detectable binding to immobilised huS2-Fc was observed for TconTS1-CD at 4 μg/mL (Fig 4A), confirming the specificity of this assay.

To investigate whether the binding of TconTS2-LD to huS2-Fc was mediated by its high-mannose *N*-glycans, huS2-Fc was treated with Endoglycosidase H (EndoH_f_), a recombinant glycosidase, which specifically cleaves high-mannose and some hybrid oligosaccharides from *N*-linked glycoproteins [42]. Released *N*-glycans were then isolated and used in a 1:2 serial dilution as potential competitive inhibitors of TconTS2-LD binding. Fig 4B shows the concentration-dependent inhibition of TconTS2-LD by the EndoH_f_ released *N*-glycans. Importantly the undiluted purified *N*-glycans inhibited binding completely, demonstrating that the interaction of TconTS2-LD with huS2-Fc is exclusively mediated by binding to *N*-glycans.

### Oligomerisation of TconTS

**Fig 5:**
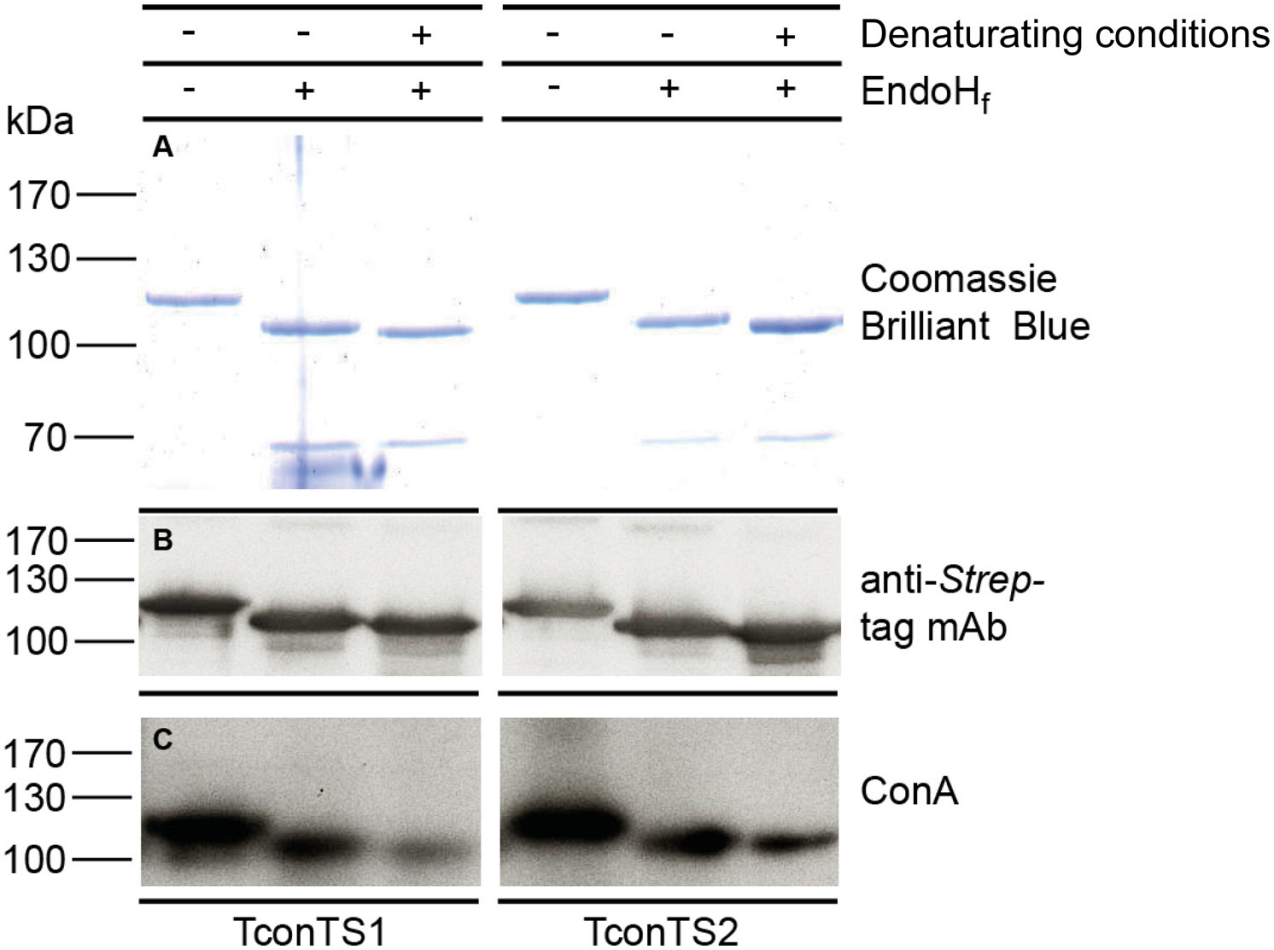
Cleavage of *N*-glycans from TconTS1 and TconTS2. 100 μg TconTS1 and TconTS2 expressed in CHO-Lec1 cells were incubated without (-) or with (+) 4000 units EndoH_f_ glycosidase under native (-) or denaturing (+) conditions as described under Methods. A: 10% SDS polyacrylamide gel with subsequent Coomassie Brilliant Blue staining. B: Western blot of deglycosylated TconTS, detected using anti-*Strep*-tag mAb. C: Concanavalin A (ConA) lectin blot using 2 μg/mL biotinylated ConA and an peroxidase conjugated avidin-biotin system (ABC-Kit, VECTASTAIN) for detection. 50 ng TconTS sample were used for ConA and Western blot analysis and 800 ng for SDS-PAGE.

**Fig 6:**
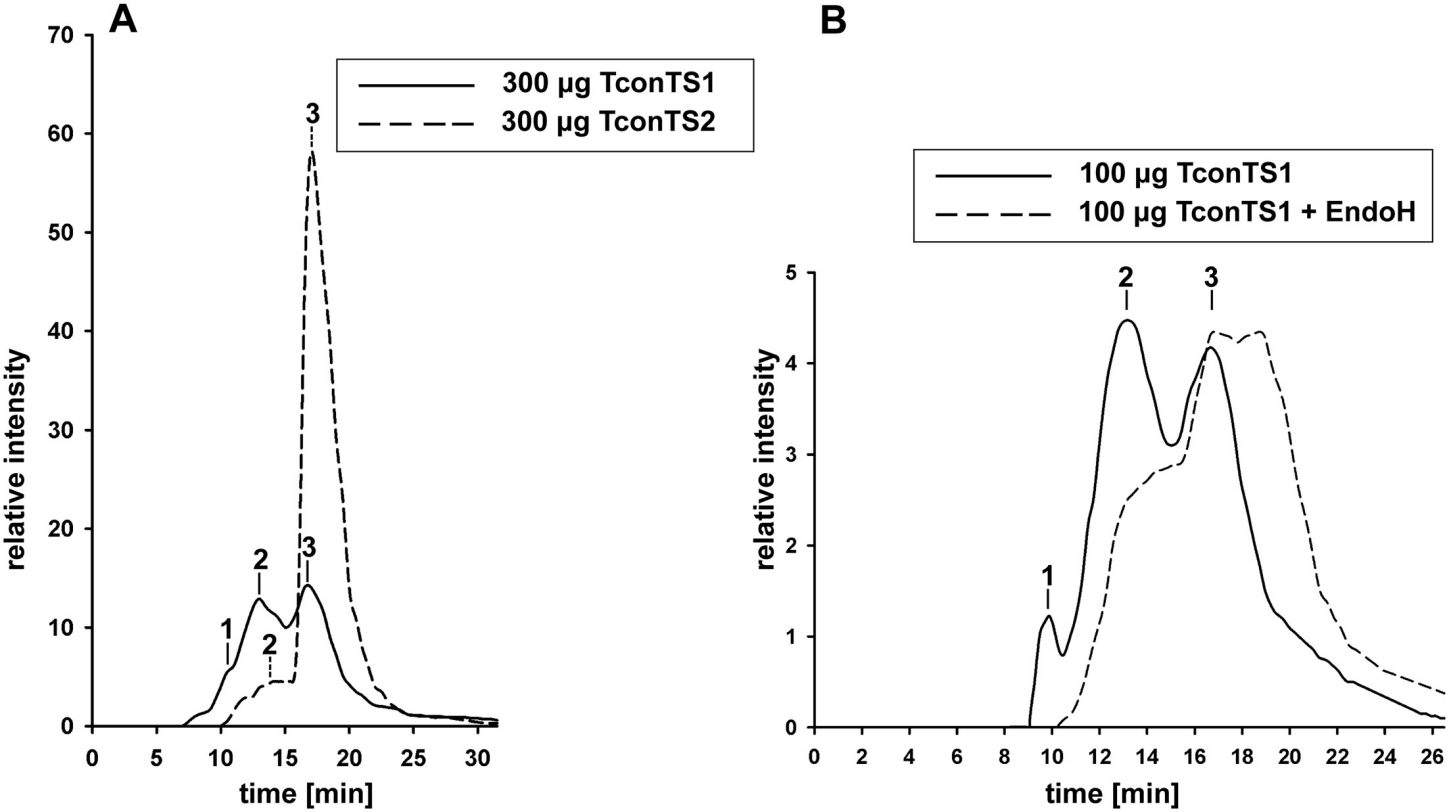
Oligomerisation of TconTS. Size exclusion chromatography on Superdex200 column and detection at E_280nm_ was used, MWs of different peaks were determined and assigned to oligomeric (1), dimeric (2) and monomeric (3) TconTS1 as described under Methods. A: Oligomerisation pattern of TconTS1 (solid line) or TconTS2 (dashed line), 300 μg protein was loaded. B: Effect of TconTS1 deglycosylation on enzyme oligomerisation. 100 μg TconTS1 were deglycosylated using 4000 units EndoH_f_ glycosidase in phosphate buffer pH 7.4 for 4 hours and directly applied to the column (dashed line). As control 100 μg TconTS1 was treated correspondingly without the addition of enzyme was loaded (solid line).

The enzymatic activities of TconTSs were previously characterised [30,31] using recombinant proteins expressed in CHO-Lec1 cells that therefore contain *N*-glycans of the high-mannose-type, similar to the recombinant huS2-Fc used here in binding/inhibition assays. *N*-glycosylation site prediction analysis revealed 8–9 potential sites in TconTSs, and none for the attached SNAP-tag (S3 Fig). In view of the interaction of TconTS-LD with huS2-Fc, we addressed the question of whether TconTS could oligomerise through binding of *N*-glycans. First, the presence of mannosylated glycans on TconTS1 and TconTS2 expressed in CHO-Lec1 cells was confirmed by lectin blot analysis using concanavalin A (ConA) (Fig 5). To analyse TconTS oligomerisation we used gel permeation chromatography of TconTS expressed in fibroblasts. These proteins contain CD and LD followed by SNAP- and *Strep*-tags, but no His-MBP at the N-terminus. Fig 6A shows the chromatograms of recombinant TconTS1 and TconTS2 under identical conditions. A double peak of similar intensities was observed for TconTS1, whereas TconTS2 showed a clear single peak with a small shoulder in front of it. The molecular weight (MW) of the TS1 peak eluting at 13 mL (peak 2) was 293 kDa and for that at 16.9 mL (peak 3) was 119 kDa (Fig 6A). This is consistent with peak 3 representing TconTS1 monomers and peak 2 dimers. Furthermore, a molecular mass of 603 kDa calculated for peak 1 eluting at 10 ml is consistent with tetramers of TconTS1. The deviation from the expected monomer (101 kDa without glycosylation) from the calculated MW (119 kDa) can be explained by increased hydrodynamic volumes (Stokes radii) of the oligomeric TconTS1, which is well-known to influence the elution behaviour of a molecule in size-exclusion chromatography [43]. Additionally, glycosylation also influences the protein elution behaviour. For the prominent peak of TconTS2 in Fig 6A eluting at 17 mL (peak 2) a MW of about 113 kDa was calculated, which is consistent with the TconTS2 monomer (100 kDa without glycosylation). The small shoulder at 13.3 mL (peak 1) that represents a MW of about 260 kDa is consistent with the dimeric form of TconTS2 (200 kDa without glycosylation). These findings strongly suggest that both TconTS1 and TconTS2 exist as monomers as well as oligomers in solution, however at different ratios. TconTS1 showed an approximate 1:1 ratio of monomer to dimer, whereas TconTS2 mainly migrates as monomer under the conditions used. To address whether oligomerisation is mediated by *N*-linked glycans, TconTS1 was enzymatically deglycosylated using EndoH_f_, and the resulting oligomeric state assessed by size-exclusion chromatography. As shown in Fig 5a clear molecular weight shift as well as a reduction in signal intensity for ConA binding was observed, strongly indicating the release of mannose containing glycans from TconTS1. Subsequent gel permeation chromatography of the deglycosylated TconTS1 resulted in a changed elution profile (Fig 6B, dashed line) compared to the untreated protein (Fig 6B, solid line). Calculating the molecular weight, peak 2 of the deglycosylated protein in Fig 6B is determined as the dimeric form with 260 kDa (elution volume: 13.3 mL) and peak 3 as the monomer with 109 kDa (elution volume: 17.2 mL). The small differences in MW can again be explained by the reduced glycosylation effect on the Stokes radius after EndoH_f_ treatment. Comparing the MW of monomer and dimer from the EndoH_f_ treated sample (peak 2: 260 kDa; 3: 109 kDa, dashed line) to those from the untreated (2: 293 kDa; 3: 119 kDa, solid line), it can be seen that both are decreased due to the loss of high-mannose *N*-glycans released by EndoH_f_. Interestingly, it was also observed that EndoH_f_ treatment of TconTS1 reduced the abundance of dimers indicated by the smaller peak 1 (dashed line) in chromatogram B compared to untreated TconTS1 (Fig 6B, solid line). In summary, these results provide strong evidence for oligomerisation of TconTS1 by binding to its *N*-glycans. Similar, but less pronounced is the oligomerisation of TconTS2.

## Discussion

**Fig 7:**
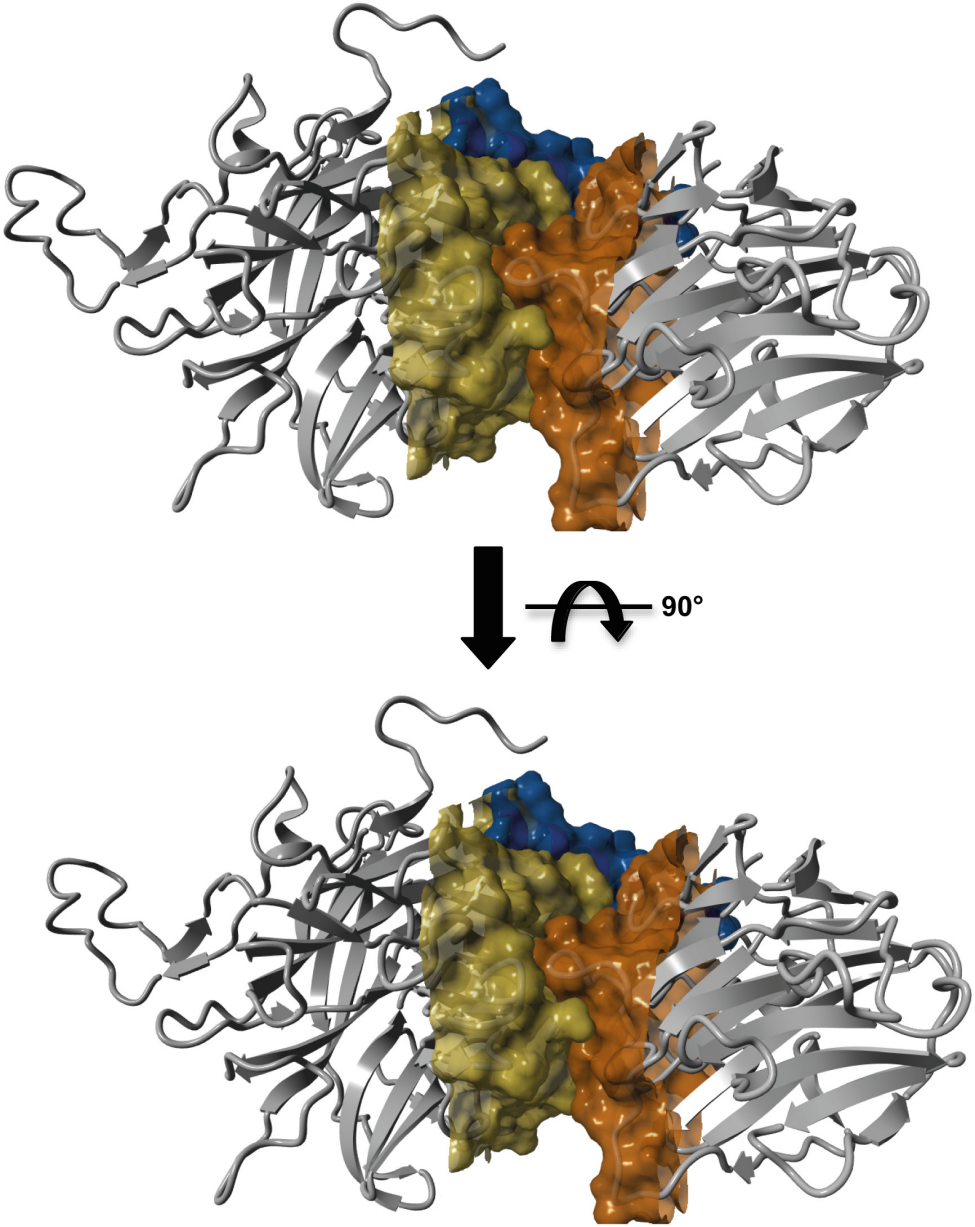
Contact site between TconTS-CD and LD. Homology model of TconTS1 was calculated using the crystal structure of TcTS (PDB: 3b69) as template and the software Yasara. Molecular surface of TconTS1 was calculated using the surface module of Yasara Structure. Illustrated are the parts of TconTS-CD (yellow) and LD (orange), which are in close contact to each other. The α-helix connecting both domains is shown in blue.

Lectin domains (LD) of the four TconTS1-4 were expressed and characterised with respect to their ability and specificity to bind carbohydrate structures. Structural comparison to other known bacterial *(Salmonella typhimurium* LT2*)* [44] and viral (*Vibrio cholerae* neuraminidase) [45] sialidases, as well as to plant lectins (*Griffonia simplicifolia* lectin 4, GS4; *Lathyrus ochrus* lectin, LOL) [22,46] provided structural evidence for potential carbohydrate-binding of TconTS-LD. Besides typical structural elements seen for several lectins, such as the β-barrel topology, each TconTS-LD also comprises a cluster of histidine, phenylalanine and arginine residues in its potential binding site, a rather shallow indentations (Fig 1C). These could presumably be involved in carbohydrate recognition via aromatic side-chain and sugar ring interaction as well as hydrogen bonding, as described for other lectins [47]. Furthermore, it is noticeable that this potential TconTS-LD carbohydrate-binding site is oriented in the same direction as the TconTS-CD catalytic site, similar to *T*. *cruzi* TS, *T*. *rangeli* SA and leech IT-sialidase [14,35,48]. This structural organisation of TconTS-CD and LD appears to be stabilised by a relatively extended close contact site between both domains comprising a network of hydrogen bonds and complementary hydrophobic patches (Fig 7).

We have identified TconTS-LD as a carbohydrate-binding domain. Employing glycan arrays, TconTS2-LD showed binding to a variety of oligosaccharides (Fig 2), whereas only a few of the glycans presented on the arrays were bound by TconTS1-LD, and none by TconTS3-LD or TconTS4-LD. It remains unclear, why only TconTS1-LD containing the α-helix at its N-terminus showed binding activity in the glycan array experiments. One explanation could be that folding or the accessibility of the binding site is compromised for TconTS1-LD without the α-helix serving as a spacer to the N-terminal MBP tag. It appears unlikely, that the α-helix is directly involved in binding activity, since the binding pattern and signal intensities for TconTS2-LD were not influenced by the presence of the α-helix. Furthermore, our binding assay data (Fig 3A) have provided evidence that TconTS1-LD also binds high-mannose glycans, which were not available on the glycan arrays, besides 1N and 4D. In addition, EndoH treatment reduced oligomerisation of TconTS1. Finally, it should also be pointed out that most of the differences in amino acid sequence among the 11 TconTS1 gene variants occur in the LD and are clustered close to the postulated binding site [30]. Therefore, it may well be possible that the other TconTS1-LD variants, besides the TconTS1a-LD used in this study, have different carbohydrate-binding specificities. Interestingly, variants that differ only in this cluster (TconTS1b and TconTS1e) have different kinetic properties for the trans-sialylation reaction, which further supports the hypothesis that TconTS-LD modulates also the enzymatic activities. This may also explain the low enzymatic activity of TconTS3 and TconTS4, since no carbohydrate-binding activity was observed for TconTS3-LD and TconTS4-LD. However, as discussed above for TconTS1-LD, TconTS3-LD and TconTS4-LD may bind to carbohydrate structures not present on the glycan arrays. In this context it should be kept in mind that the amino acid sequence diversity of TconTS-LDs (S2 Table) is high enough to allow different binding activities. Furthermore, the structures of spacers used to immobilise the glycans may have prevented binding of TconTS3-LD and TconTS4-LD, since these affect lectin binding to glycan arrays [49,50]. However, given the diverse spacer structures and lengths utilised here, it appears less likely that binding would have escaped detection. Finally, we cannot exclude the possibility that insufficient folding of TconTS-LDs in the bacteria during expression is the reason for undetected lectin activity.

According to a phylogenetic analysis comparing separately TconTS-LDs and TconTS-CDs [31] TconTS1-LD and TconTS2-LD are more closely related with each other than TconTS3-LD and TconTS4-LD. However, when comparing CDs, TconTS2-CD is more closely related to TconTS3-CD and TconTS4-CD, but least to TconTS1-CD. Interestingly, the highest enzyme activities of TconTS were found with TconTS1 and TconTS2, whereas TconTS3 and TconTS4 were 100- or 1000-fold less active [31]. Together with the carbohydrate-binding activities of TconTS1-LD and TconTS2-LD described in this study, it can be postulated that LDs may directly influence enzyme activities. In agreement with this hypothesis is the observation that disrupting a salt bridge between R642 and E648 in the LD of *T*. *cruzi* TS enhanced trans-sialidase relative to sialidase activity [35,51]. Along this line it could be postulated that binding of oligosaccharide substrates to TconTS via oligomannose clusters may lead to an improved presentation of the terminal acceptor galactose residues towards the active centre of the CD and therefore lead to an enhanced catalytic efficiency.

Ammar et al. recently demonstrated that TconTS can induce activation of endothelial cells [36] and assumed that the TconTS-LD may be involved in this process. In this context, they prepared a mutant (Y438H) of TconTS1 (TcoTS-A1 according to their nomenclature) to prevent transfer activity. Since this mutant competed with Mal (*Maackia amurensis* lectin) binding to α2,3-linked Sia of the surface of endothelial cells, they concluded that lectin binding to cell surface carbohydrates play the key role in endothelial cell activation. Based on our data regarding the carbohydrate-binding specificities of TconTS-LD, it appears unlikely that the LD mediates the activation of endothelial cells. However, their findings are in agreement with an involvement of the CD.

TconTS2-LD binds structures containing mannose, such as α1-6-mannobiose and α1–3,α1-6-mannotriose (Fig 2) and several galactose and lactose containing oligosaccharides. Given that the few fucosylated and sialylated glycans recognised by TconTS2-LD all possess core galactose, lactose or *N*-acetyl-lactoseamine units, it can be assumed that fucosylation and/or sialylation at least in some positions does not interfere with binding. For example, when fucose is linked to positions of the glycan, which is more solvent exposed, it will not disturb ligand binding.

Binding of TconTS-LD to galactosyl, lactosyl and potentially also sialyl glycans was not completely unexpected, since they also serve as substrates for TconTS [6,30,31] and lectin-like binding to sialidase substrates is not uncommon. For example, the lectin-like domain of *Vibrio cholerae* sialidase (VCS) binds *N*-acetylneuraminic acid in a similar manner compared to the catalytic domain but without hydrolytic activity [52].

Engstler et al. 1995 investigated procyclic TconTS substrate specificity using a selection of sialylated donor substrates and galactosylated acceptor substrate oligosaccharides, including the monosaccharide mannose, as substrates for TS [5]. Whereas transfer of Sia to terminal galactose oligosaccharides and even the monosaccharide galactose was observed, mannose did not appear to be a suitable acceptor for sialic acid transfer. Thus, our discovery that TconTS-LD binds to mannosylated oligosaccharides suggests a yet unknown function of the LD, distinct from that of the CD. The overall ligand specificities of TconTS-LD, binding to both mannosylated as well as galactosylated glycans, but not to glucose containing oligosaccharides (Figs 2 and S2), differs from that of other mannose-specific lectins, such as concanavalin A (ConA, *Canavalia ensiformis*) [53,54], LOL (*Lathyrus ochrus* lectin) [24] or GNA (*Galantus nivalis agglutinin*, Snowdrop lectin) [23]. In contrast to mannose and glucose, which both show equatorial orientation of the C4-OH group, in Gal the orientation of C4-OH is axial, which does not support binding to ConA or LOL, since C4-OH is involved in carbohydrate recognition by these lectins. Similarly, GNA specifically binds Man via an essential hydrogen bond to its C4-OH group. Considering these ligand selection mechanisms, it appears more likely that two structurally independent binding sites provide TconTS2-LD binding to both mannose- and galactose-containing oligosaccharides. This hypothesis is supported by our STD NMR data that clearly indicate that lactose and α1–3,α1-6-mannotriose do not compete for the same site on TconTS2-LD. According to published STD NMR data for lactose [55] and α1–3,α1-6-mannotriose [56], it could be concluded that both moieties of lactose interact with TconTS2-LD, at least partially. For example, this is indicated by the βGlc-H2 and βGal-H4 protons of lactose (Fig 3B). All signals observed in the off-resonance spectrum of α1–3,α1-6-mannotriose could also be identified in the STD NMR spectra, showing no clear preference for any proton. This indicates specific binding and that all three mannose units appear to be in close contact with the protein. A similar binding epitope of α1–3,α1-6-mannotriose has recently been described for the antibiotic Pradimicin S [56]. The binding epitope for lactose is less uniform, suggesting that not all protons of the disaccharide are in the same close contact with the protein. The exception constitutes the βGlc-H2-proton, for which a two-fold higher STD signal intensity was observed compared to several other protons with similar STD effects than most of the protons of α1–3,α1-6-mannotriose (Fig 3D). This finding might be explained by selective interaction of the βGlc configuration of lactose with TconTS2-LD. An important result was that the STD NMR effects of both oligosaccharides were independent of the presence of the other ligand, since they were identical, if TconTS2-LD was incubated with an equimolar mixture, to those obtained for the individual compounds. If they did compete for the same site, reduced STD NMR signals would have occurred for either both ligands, if they bind with similar affinities, or at least for that ligand, which binds with much lower affinity, if they bind with different affinities [40]. It can be excluded that the STD NMR signals of lactose or α1–3,α1-6-mannotriose reflect interactions with the MBP tag, since no binding of MBP to these structures have been observed in our glycan array experiments, which is in agreement with previous studies on MBP specificity applying diverse methods [57,58] including STD NMR [59]. In conclusion, our findings suggest that two distinct binding sites exist on TconTS2-LD, similar to the lectins WGA [60] and GNA [23].

Interestingly, in a previous STD NMR study TcTS binding to lactose was only observed in the presence of Neu5Ac [16]. Apparently, TcTS and TconTS2-LD have different carbohydrate-binding activities, since TconTS2-LD clearly binds to lactose and α1–3,α1-6-mannotriose in the absence of Sia. Furthermore, the binding epitope for lactose in complex with TconTS2-LD is distinct from that observed for TcTS [16], most pronounced is this difference for the STD NMR signal of βGlc-H2, which was not observed for TcTS. This further underlines that the binding site for lactose on TconTS2-LD is distinct from the acceptor-binding site of the CD described for TcTS.

The crystal structure of TcTS [14] revealed that the binding pocket of the catalytic domain is located at the same side as carved β-barrel groove of the lectin domain, in which conserved histidine and tyrosine residues were identified, known to be involved in carbohydrate recognition of other lectins [47]. Therefore, we assume this position to be the potential carbohydrate-binding site on TconTS-LDs. Furthermore this hypothesis is in agreement with our data assuming TconTS-LD interacts simultaneously with more then just one monosaccharide, suggesting an extended binding site, also termed sub-site multivalency [61]. However, detailed structural studies, such as X-ray crystallography of TconTS2-LD with these oligosaccharide ligands are required to locate and investigate these binding sites precisely.

For several lectins it has been reported that oligomerisation enhances binding due to interactions of multivalent glycoconjugate ligands to multiple binding sites of oligomeric lectins [47,62,63]. Also for TconTS1-LD and TconTS2-LD multivalent interactions in larger complexes strengthen binding to the target glycoprotein, since in our binding/inhibition assays pre-complexing of TconTS-LD with the anti His-tag mAb and the corresponding secondary antibodies used for detection lead to stronger signals compared to applying every component in consecutive steps (S4A and S4B Fig).

By large, this binding is mediated by the high-mannose *N*-glycans of huS2-Fc, since it could be inhibited with *N*-linked oligosaccharides, enzymatically released from recombinant huS2-Fc, as competitive inhibitors. Interestingly, for the monosaccharide α-methyl-mannopyranoside, which is a well-known inhibitor for ConA [64], only slight inhibition of TconTS-LD binding could be observed at 50 mM (S4C Fig). This evidence for binding towards complex oligosaccharide ligands was already reported earlier for several other lectins, including the lectin GS4 (*Griffonia simplicifolia* lectin IV), which shows high affinity to poly- but not to monosaccharides [47,65]. This mechanism, providing poor affinities for lectins to monosaccharides, prevents unspecific interference from competing, structurally similar molecules and enhances ligand selectivity. In addition, recombinant TconTS enzymes expressed in CHO-Lec1 cell lines contain these *N*-linked high-mannose-type oligosaccharides. Eight to nine potential *N*-linked glycosylation sites are found in recombinant TconTS, distributed over CD and LD. As expected, lectin blot analysis using ConA, for detection of mannose oligosaccharides, clearly confirmed the presence of *N*-linked mannosylated glycans on recombinant TconTS expressed in CHO-Lec1 cells (Fig 5). Recombinant TconTS exhibit much higher enzyme activity, if expressed in these fibroblasts compared to those expressed in bacteria (S4D Fig). This is possibly related to the absence of *N*-glycosylation in bacteria, which may have an indirect effect on enzyme activity by influencing proper enzyme folding or even directly by glycan mediated TconTS oligomerisation. In agreement with the latter scenario, binding to *N*-linked mannosyl oligosaccharides of recombinant TconTS expressed in CHO-Lec1 cells leads to oligomerisation. This conclusion is supported by (1) the observation that TconTS1 elutes in about equal amounts as monomer and as dimer in size exclusion chromatography (Fig 6A) and (2) that the removal of *N*-linked glycans with EndoH_f_ glycosidase leads to a clear shift from the dimer towards the monomeric form of TconTS1 (Fig 6B). The remaining dimers are likely to be due to inefficient deglycosylation, as suggested by ConA lectin blot analysis (Fig 5) or due to other carbohydrate-independent protein-protein interactions. It remains unclear as to why TconTS1 oligomerises to a larger extent than TconTS2. One possible explanation may be differences in the glycosylation pattern of the two recombinant enzymes. That is, potential *N*-glycosylation sites are distributed differently in both enzymes with nine potential sites for TconTS1, eight for TconTS2, only one site being conserved. Finally, it should be mentioned that it is also unclear, which of the predicted *N*-glycosylation sites of recombinant TconTS utilised in this work are actually glycosylated. In addition, it should be noted that the glycosylation pattern of the native TconTS is unknown as well.

Based on the finding that TconTS-LD binds to high-mannose-type *N*-glycans we assumed that glycoconjugates containing high mannosylated structures might be preferred natural acceptor substrates for TconTS. Interestingly, such high-mannose-type glycans have been identified on the surface of *T*. *congolense* in both, bloodstream (mammalian host) and procyclic (tsetse vector) forms, either linked to amino acids or as part of the GPI anchors [66–69]. The African parasites express two major stage specific, glycosylphosphatidylinositol (GPI)-anchored glycoproteins on their surfaces, the variant surface glycoprotein (VSG) of the bloodstream form and procyclins of the procyclic form. During development of the bloodstream form (BSF) in the mammalian host, the parasites express a surface coat composed of hundreds of immunologically distinct VSG molecules (antigenic variation) to evade host immune response. These VSGs share relatively little primary sequence homology [70] but are structurally related to each other [71]. It has been demonstrated that VSGs from *T*. *congolense* and *T*. *brucei* BSF are highly glycosylated, exhibiting glycan structures similar to those of higher eukaryotic *N*-linked oligosaccharides. Interestingly, they are composed of *N*-linked high-mannose-type oligosaccharides (Man_5-9_) and *N*-acetyllactosamine oligosaccharides, as well as branched poly-*N*-acetyllactosamine oligosaccharides with a Man_3-4_ core (GalGlcNAc)_3_Man_3_GlcNAc, which were also found to be sialylated in *T*. *congolense* [72–74]. The fact that terminal Sia were found on VSGs indicate that these are substrates for TS present on the cell surface. In this context, it is plausible, that TconTS-LD contributes to the binding of *T*. *congolense* VSG to TconTS via oligomannose oligosaccharides present on these glycoproteins.

When parasites are taken up by tsetse fly through a blood meal, VSGs are replaced by procyclic stage specific, membrane bound, major surface proteins known as procyclins or procyclic acidic repetitive proteins (PARP) [75,76] in *T*. *brucei* and glutamic acid/alanine-rich protein (GARP) in *T*. *congolense* [66–68,77]. Interestingly, compared to the highly *N*-glycosylated *T*. *brucei* BSF VSGs, procyclins only contain a single *N*-glycosylation site, substituted with oligomannose oligosaccharide Man_5_GlcNAc_2_, which is unusual and rare, but not unique [78]. The primary sequence of *T*. *congolense* procyclic GARP does not contain a single potential *N*-glycosylation site, which was also experimentally confirmed, as well as the absence of conventional *O*-linked glycans [77]. However, two large Man and Gal-rich oligosaccharides of the type Man_11_Gal_6-7_ linked via phosphodiester bonds to two threonine residues were found [77]. Considering these findings, GARP may also be a potential binding partner for TconTS-LD. Indeed, TconTS-mediated sialylation of GARP has been demonstrated and even procyclin was equally efficiently sialylated by the same enzyme, indicating their functional similarities at least as substrates for TS [5]. However, sialylation of procyclin occurs at the glycan moiety of its GPI anchor [26], which has been structurally characterised [79–81]. They share the common core structure of GPI anchor EtNH_2_-HPO_4_-6Man(α1–2)Man(α1–6)Man(α1–4)GlcNH_2_(α1–6)-PI, but with an additional glycosylation at the Man_3_-core, which is unique for African trypanosomes [80,81]. It comprises oligolactosamine oligosaccharides (Gal-GlcNAc)_9_ for *T*. *brucei* procyclin GPI anchors and oligogalactosyl oligosaccharides Gal_5-7_ for *T*. *congolense*, which both represent substrates for trans-sialylation [77,80]. In this context it is important to note that GARP was co-purified and co-immunoprecipitated with TS-form 1 from procyclic cultures of *T*. *congolense* [6], indicating a relatively strong interaction between these two surface proteins. It is in complete agreement with the data of this study that this interaction is mediated by TconTS-LD binding to the GPI anchor Man_3_-core of GARP, since binding to similar oligomannose oligosaccharides like Man(α1–3)Man and Man(α1–6)Man was observed by glycan array and STD NMR analysis. Homology models of TconTS1-4 revealed, that the distance between the catalytic tyrosine residue in the active centre of TconTS-CD and a conserved phenylalanine residue in the predicted TconTS-LD carbohydrate-binding site, ranges from 40.5 to 42.6 Å. With an average diameter of 7 Å for a single hexose unit, an oligosaccharide of minimum 6 monosaccharide units from the Man_3_-core is needed to reach the catalytic centre of TconTS-CD, depending on the glycosidic linkage of the oligosaccharide. Consistent with this, the oligosaccharide Gal_5-7_ of the GARP GPI-anchor has the appropriate size to reach the catalytic centre when TconTS-LD is bound to the Man_3_-core. In this case both TconTS domains could interact simultaneously with different sections of the same oligosaccharide, whereby the binding affinities of each domain contribute to the overall TconTS binding affinity, which is then clearly enhanced, consistent with the observed co-purification of TS-form 1 with GARP from *T*. *congolense* cultures [6]. This would be somewhat similar to the situation reported for the *Vibrio cholerae* sialidase, where Neu5Ac binds to a lectin domain without hydrolytic activity, leading to an increased affinity of the enzyme for highly sialylated regions [52]. In addition, the apparent organisation of the two native TS-forms isolated by Tiralongo *et al*. [6], who observed TS-form 2 as dimers and TS-form 1 as oligomers is in very good agreement with the oligomannosyl oligosaccharide-mediated interaction of recombinant, high-mannosylated TconTS1 and TconTS2. It should be noted that these are not identical to purified TS-forms 1 and 2 described by Tiralongo *et al*. [6]. Furthermore, *T*. *brucei* TS has been previously purified by ConA affinity chromatography from procyclic trypanosomes, suggesting that also this TS is highly mannosylated in its native state on the parasite [27].

In summary, we identified TconTS1-LD and TconTS2-LD as a carbohydrate recognition domain (CRD), exhibiting different binding affinities to several oligogalactosyl, oligolactosamine and oligomannosyl glycans, via two independent binding sites. Functionally, the interaction with specific oligomannosyl structures appears to be required to facilitate TconTS oligomerisation, and binding to oligogalactosyl and oligolactosamine oligosaccharides may represent the recognition event associated with TS acceptor substrate binding. Since the LD from *T*. *congolense*, *T*. *brucei*, *T*. *cruzi* and potentially other trypanosomes are structurally related, this may be a general function of TS-LD and may open new avenues for the design of novel inhibitors for therapeutic applications controlling trypanosomiasis in Africa and Latin America.

## Methods

### Materials

All chemicals and reagents used in this study were analytical grade. Recombinant EndoH_f_ glycosidase (EndoH_f_) was from New England Biolabs, UK. *Pfu* and *Taq* DNA polymerase, *HindIII*, *NcoI*, *NotI*, *SalI* Fast Digest restriction enzymes, T4-DNA ligase, isopropyl-β-D-1-thiogalactopyranoside (IPTG), Dithiothreitol (DTT), Coomassie Brilliant Blue (Page Blue), protein molecular weight marker (PageRuler), GeneJET DNA Gel Extraction Kit, BCA Protein Assay Kit, enhanced chemiluminescence system (ECL-Kit), fluorescein diphosphate tetraammonium salt (FDP), Luria Broth (LB) microbial growth medium, anti His-tag mouse polyclonal antibody, anti-mouse-IgG-alkaline phosphatase-conjugated donkey polyclonal antibody (serum purified) were from Thermo Scientific, Germany. Biozym LE Agarose was from Biozyme Scientific, Germany. StrepTactin Sepharose, purification buffers and anti *Strep*-tag rabbit polyclonal antibody were from IBA, Germany. Anti-mouse-IgG-TexasRed conjugated rabbit polyclonal antibody, anti-rabbit-IgG-TexasRed conjugated donkey polyclonal antibody were purchased from Life Technologies. β-D-galactosyl-(1–4)-α-D-glucose (4α-lactose), β-D-galactosyl-(1–4)-α-D-*N*-acetylglucosamine (4α-*N*-acetyllactosamine), 4-α-D-maltose, α-D-glucopyranosyl-(1–4)-α-D-glucose (4α-maltose), α-D-glucopyranosyl-(1–4)-α-D-glucopyranosyl-(1–4)-α-D-glucose (4α-maltotriose), α-methyl-D-mannose, α-D-mannosyl-(1–2)-D-mannose (2α-mannobiose), α-D-mannosyl-(1–3)-D-mannose (3α-mannobiose), α-D-mannosyl-(1–4)-D-mannose (4α-mannobiose), α-D-mannosyl-(1–6)-D-mannose (6α-mannobiose), α-D-mannosyl-(1–3)-[α-D-mannosyl-(1–6)]-D-mannose (3α,6α-mannotriose), polyethylene glycol sorbitan monolaurate (TWEEN 20), Gel Filtration Markers Kit for protein molecular weights between 29,000–700,000 Da were from Sigma-Aldrich, Germany. Concanavalin A (ConA), Sepharose and biotinylated recombinant ConA were purchased from Galab, Germany. VECTASTAIN ABC detection system was from Vector laboratories, UK. Ultrafiltration units Vivacell and Vivaspin6 were from Sartorius, Germany. X-ray film was purchased from GE Healthcare, Sweden. Protino Ni-NTA Agarose and NucleoBond Midi Plasmid DNA Purification Kit were from Macherey-Nagel, Germany. Polyvinylidenedifluoride (PVDF) membranes were from Millipore, Germany. 96-well transparent microtitre plates were from Sarstedt, Germany. High binding 384-well black microtitre plate were purchased from Corning, USA. 6 mL gravity flow columns were from Biorad, Germany.

### Cloning and expression of recombinant TconTS-LD

To obtain non-glycosylated TconTS-LD as recombinant proteins in sufficient amounts, a bacterial expression system based on a modified pET28a+ expression vector was established. Modifications made comprised an N-terminal poly-histidine-tag (His-tag) followed by maltose binding protein (MBP) and a tobacco etch virus (TEV) protease cleavage site [82]. MBP was used to enhance expression and solubility of TconTS-LD in *E*. *coli*. SNAP- Strep- and His-tags were employed for affinity purification, detection and immobilisation of recombinant protein as previously described [30,31] The DNA sequence encoding the His-MBP part was amplified form pETM–41 (EMBL, Germany) using *Pfu* DNA polymerase and the appropriate primers (S3 Table). The purified PCR products were ligated into the *NcoI* and *SalI* digested pET28a (Novagene, USA) bacterial expression vector and transformed into chemical competent *E*. *coli* BL21 (DE3)(BD Bioscience, Clonetech, USA). Sequence identity was confirmed by commercial sequencing at the Max Planck Institute for Marine Biology (MPI) Bremen and results were evaluated using the Geneious Software.

The modified eukaryotic expression pDEF-based vectors coding for TconTS1, TconTS2, TconTS3 and TconTS4 [30,31] were used as template to amplify TconTS1-LD, TconTS2-LD, TconTS3-LD and TconTS4-LD containing C-terminal SNAP and Strep tags. Two sets of sense primers were designed for each TconTS variant (S3 Table). The same reverse primer including a *NotI* restriction site (underlined) was used for all TconTS-LD constructs, since all TconTS-LD constructs contain C-terminal Strep-tag. Purified PCR-products were ligated in frame into the *HindIII* and *NotI* digested, modified pET28aMBP vector and transformed into *E*. *coli* Rosetta (DE3) pLacI (BD Bioscience, Clonetech, USA). Plasmid preparations of pET28aMBP/TconTS-LD were prepared and characterised as described above.

*E*. *coli* Rosetta (DE3) pLacI colonies freshly transformed with pET28aMBP/TconTS-LD were inoculated in 20 mL of 50 μg/mL kanamycin containing Luria Broth (LB) medium and incubated overnight at 37°C and 240 rpm shaking. 2 mL of these overnight cultures were transferred into 1 L of 50 μg/mL kanamycin containing LB medium and grown at 37°C and 240 rpm until an optical density at 600 nm of 0.5 was reached. Recombinant protein expression was then induced by the addition of isopropyl-β-D-thiogalactopyranoside (IPTG), with a final concentration of 0.1 mM and cells were incubated for additional 120 min at 37°C and 240 rpm. Cells were harvested by centrifugation for 15 min at 1500 x g, 4°C and the pellet was resuspended in 20 mL lysis buffer 50 mM NaH_2_PO_4_, pH 8.0, 300 mM NaCl. Lysis was done by ultrasonication on ice applying 9 pulses of 20 sec each (50 Watts) with 10 sec pauses between pulses. The bacterial lysates were centrifuged for 30 min at 15000 x g, 4°C. Clear supernatants were transferred to 4 mL of equilibrated Ni-NTA beads and incubated on a rotation wheel (6 rpm) at 4°C for 120 min. The suspensions were transferred to 6 mL gravity flow columns in portions, until all beads were settled in the column. Beads were washed with 40 mL wash buffer containing 50 mM NaH_2_PO_4_, pH 8.0, 150 mM NaCl, 20 mM imidazole. Recombinant TconTS-LD was eluted using 250 mM imidazole in 50 mM NaH_2_PO_4_, pH 8.0, 150 mM NaCl and directly applied to a new gravity flow column containing 1.6 mL StrepTactin beads equilibrated with wash buffer (100 mM Tris-Cl, pH 8.0, 150 mM NaCl and 1 mM EDTA) and beads were washed with 5 column volumes of wash buffer. Recombinant proteins were eluted with wash buffer containing 2.5 mM desthiobiotin and dialysed against 10 mM phosphate buffer, pH 7.4 using a Vivaspin6 filtration unit with a 100 kDa cut off. Purified TconTS-LD was characterised by SDS-PAGE and Western blot analysis and quantified by BCA assay using bovine serum albumin (BSA) as standard.

*T*. *congolense* recombinant TconTS1 and TconTS2 containing catalytic (CD) and lectin domain (LD) expressed by CHO-Lec1 cells were prepared from culture supernatants and characterised using SDS-PAGE, Western Blot and BCA assay analysis as described [30].

### Glycan array

Glycan arrays consisting of 367 diverse glycans with and without the presence of one of three spacers (sp2, sp3 or sp4 [49]) were prepared from two previously described glycan libraries [83,84]. Amine containing glycans with spacer’s sp2, sp3 or sp4 were synthesised as previously described [49] and glycans without spacers were amine functionalised as previously published [85]. All glycans were suspended in 1:1 DMF:DMSO at a concentration of 500 mM and were printed onto SuperEpoxy 2 glass slides (ArrayIt, Sunnyvale, CA) using a ArrayIt SpotBot Extreme array spotter in a six pin subarray print per glass slide format. All glycans were printed in replicates of four, including four FITC control spots as well as additional position controls (S1 Fig), per subarray using SMP4 pins and a contact time of 1 second at 60% relative humidity, with pins being reloaded after every 12 spots.

Prior to performing glycan array experiments, slides were scanned using a ProScanArray Microarray 4-laser scanner (Perkin Elmer, Waltham, MA) using the blue argon 488 laser set to the FITC settings (492 nm excitation and 517 nm emission). Array slides were blocked with 0.1% BSA in 50 mM phosphate buffered saline (PBS), pH 7.4 for 5 min at 22°C. After washing with PBS, each slide was dried by placing them in an empty 50 mL tube and centrifuging for 5 min at 200 x g (900 rpm). Recombinant TconTS-LD (2 μg) was incubated at a molar ratio of 1:2:3 with anti His-tag mouse polyclonal antibody (10 mg/mL, Cell Signalling Technology), anti mouse-IgG-Alexa555 conjugated rabbit polyclonal antibody (2 mg/mL, Life Technologies) and anti rabbit-IgG-Alexa555 conjugated goat polyclonal antibody (2 mg/mL, Life Technologies) in 50 mM PBS, pH 7.4 containing 0.1% BSA and 10 mM maltotriose for 15 min on ice protected from light. All subarrays on the slide were isolated using a Gene Frame (1.5 x 1.6 cm, 65 μL, Abgene, Epsom, UK) prior to the addition of the TconTS-LD-antibody mix to the array. A coverslip was applied to the GeneFrame and array slides incubated for 30 min at 22°C in the dark. The GeneFrame and coverslip were subsequently removed and the slide gently washed twice with 50 mM PBS, pH 7.4 containing 0.01% TWEEN 20 and 10 mM maltose, and once with 50 mM PBS, pH 7.4 containing 10 mM maltose. Slides were dried by centrifugation for 5 min at 200 x g (900 rpm), allowed to air dried for a further 5 min, and the fluorescence associated with the array spots detected using the microarray scanner settings outlined above. Image analysis and spot visualisation was performed using the ProScanArray software, ScanArray Express (Perkin Elmer). The resulting images were visually examined. Fluorescence signals were judged as being positive, if all four replicates for a glycan were clearly detectable (S1 Fig).

### STD NMR

TconTS2-LD was several times buffer exchanged to 10 mM deuterated phosphate buffer, pD 7.4 using a Microcon centrifugal ultrafiltration device (cut off 10 kDa). 200 μL of a solution containing 5.5 μM TconTS2-LD was prepared for each experiment. 1024 Scans per STD NMR experiment were acquired as described before [40]. In separate experiments, lactose and 3α,6α-mannotriose were added to the TconTS2-LD solution resulting in 3.45 mM (for lactose) or 1.73 mM (for 3α,6α-mannotriose or in the mixture of both oligosaccharides) final concentrations. The STD NMR spectra were obtained by subtracting the on- from the off-resonance spectra. As controls, STD NMR spectra for only TconTS2-LD or ligand were recorded under identical conditions used. Data acquisition and evaluation was performed using NMR software TopSpin 3.2 (Bruker Daltonics, Germany).

### TS-LD binding/inhibition assay

Microtitre plate based binding and inhibition assays, used for characterising protein binding to sialylated glycoproteins, has been described for siglecs [86]. In this study a modified version of these assays was established to investigate TconTS-LD binding to mannosylated glycoproteins. Recombinant huS2-Fc expressed and purified as previously described [87] was used as binding partner for TconTS-LD, since it contains high-mannose *N*-glycans due to expression in CHO-Lec1 cells. 5 μL of 5 μg/ mL huS2-Fc in 50 mM NaHCO_3_, pH 9.6, were immobilised on a high binding 384-well microtitre plate (Corning, USA) overnight at 4°C. The plate was washed five times with 20 μL 10 mM Tris-Cl, pH 7.5, 150 mM NaCl containing 0.05% Tween20 (TBS-T) per well. A 1:2 serial dilution of TconTS2-LD ranging from 4–0.125 μg/ mL in 10 mM TBS-T was prepared. 0.2 μg/ mL anti His-tag mouse polyclonal antibody and 0.2 μg/ mL anti mouse-IgG alkaline phosphatase(AP)-conjugated donkey polyclonal antibody was added to each dilution step and incubated on ice for 30 min. 5 μL of each sample were transferred in triplicates onto the washed microtitre plate and centrifuged for 1 min at 600 x g. The plate was covered with parafilm and incubated at 4°C for additional 3.5 hours. After washing the plate 4 times with 10 mM TBS-T and twice with 10 mM TBS, 20 μL of freshly prepared fluorescein diphosphate (FDP) substrate solution (50 mM Tris-Cl, pH 8.5, 10 mM MgCl_2_, 20 μM FDP) was added to each well and the kinetic fluorescence measurement was immediately started employing a Tecan Infinite F200 Pro microtitre plate reader (Tecan, Germany). As controls, wells containing and lacking immobilised huS2-Fc were incubated with both antibodies but in the absence of TconTS2-LD. For comparison, 4 μg/ mL recombinant catalytic domain (CD) of TconTS2 was used instead of TconTS2-LD under the same conditions used.

Inhibiton assays were performed following the same procedure as for the binding assay, but free oligomannose *N*-glycans were added as potential competitive inhibitors during the incubation with TconTS2-LD. Oligomannose *N*-glycans were released by endoglycosidase H (EndoH_f_) treatment of 10 μg huS2-Fc in 50 mM sodium citrate, pH 5.5 at 37°C for 4 hours. Proteins were acetone precipitated at -20°C overnight [30]. Following centrifugation the supernatant was transferred into a fresh reaction tube and solvent was removed using SpedVac evaporator for 1 hour at 30°C, 100 mbar. Glycans were resuspended in 10 mM TBS and used in inhibition assay as 1:2 dilution series. Data acquisition was done using the software Magellan 7.2. Binding and inhibition curves were generated using SigmaPlot 11.

### Deglycosylation of TconTS

TconTS1 and TconTS2 were enzymatically deglycosylated using recombinant EndoH_f_ cleaving the chitobiose core (GlcNAc(1–4)-β-GlcNAc) of high-mannose *N*-glycans from glycoconjugates [88]. In brief, 500 μL 10 mM phosphate buffer, pH 7.4 containing 100 μg TconTS and 4000 units EndoH_f_ were incubated for 4 hours at 37°C. For deglycosylation of TconTS under denaturing conditions, 100 μg TconTS were incubated in 20 μL denaturing buffer containing 40 mM dithiothreitol (DTT) and 0.5% sodium dodecyl sulfate (SDS) for 10 min at 95°C. After the addition of sodium citrate, 50 mM final concentration, pH 5.5 and 4000 units EndoH_f_, reaction mix was incubated for additional 60 min at 37°C. *N*-deglycosylation efficiency was determined by ConA lectin blot analysis.

### Gel permeation chromatography

Oligomerisation of TconTS was analysed employing a fast protein liquid chromatography (FPLC) system (Amersham Pharmacia, USA) using Superdex 200 10/300 GL (GE Healthcare, Sweden) size exclusion column and photometric detection at 280 nm. Chromatographic analysis were done at 4°C. In brief, column was equilibrated with 10 mM phosphate buffer pH 7.4 and calibrated using a gel filtration marker kit for protein molecular weights between 29,000–700,000 Da (Sigma-Aldrich, Germany) according to manufactures instructions. 100 to 300 μg TconTS in 500 μL sample volume were injected and separated at a flow rate of 0.5 mL/min. Absorbance at 280 nm was continuously recorded through an analog writer and subsequently transformed to digital chromatograms using the software SigmaPlot 11. EndoH_f_ treated samples were analysed in the same manner.

### Western blot and Con A lectin blot analysis

Protein samples were separated employing SDS-PAGE as described previously [89] using a MiniProtean III electrophorese Unit (Bio-Rad, Germany) and stained with Coomassie Brilliant Blue.

Western blot analysis were performed as previously described [30], using primary anti S*trep*-tag rabbit antibody (1:1000) and secondary anti rabbit-IgG donkey horseradish peroxidase(HRP)-conjugated antibody (1:40000). Membranes were developed using enhanced chemiluminescence system (ECL-Kit, Thermo Scientific, Germany) and X-ray film (GE Healthcare, Sweden).

ConA lectin blots were performed similar to the procedure for Western blotting. Instead of applying primary antibody, 2 μg/mL solution of biotinylated recombinant ConA in 10 mM phosphate buffer, pH 7.4 was added to the membrane and incubated overnight at 4°C. Avidin-biotin HRP conjugated system (VECTASTAIN ABC-Kit, Vector Labs, UK) was used for detection according to manufactures instructions.

### Homology modelling

Homology models of TconTS-LD containing or lacking the α-helix were calculated employing the molecular modelling software Yasara 13.3.26 [90–95] as previously described [30]. In brief, crystal structure of *Trypanosoma cruzi* TS (PDB: 3b69) [14] was used as a template structure for calculating the models. Yasara *homology modelling* module were modified manually from the default settings of the program as follows: Modelling speed: slow, PsiBLASTs: 6, EValue Max: 0.5, Templates total: 1, Templates SameSeq: 1, OligoState: 4, alignments: 15, LoopSamples: 50, TermExtension:10. The molecular and electrostatic potential surfaces were calculated using the ESPPME (Electrostatic Potential by Particle Mesh Ewald) method of Yasara *Structure* with the following parameters: Force field: AMBER96, Algorithm used to calculate molecular surface: numeric, Radius of water probe: 1.4 Å, Grid solution: 3, Maximum ESP: 300 kJ/mol. Red colour indicates a positive potential, blue a negative and grey a neutral.

## Supporting Information

S1 Fig Identification of potential TconTS-LD oligosaccharide ligands. Purified TconTS-LDs (2 μg) were pre-complexed with mouse anti-His, rabbit anti-mouse TexasRed, and donkey anti-rabbit TexasRed and applied to glycan array slides in the presence and absence of 10 mM maltotriose. Following washing and drying array slides were scanned using the ProScanArray Microarray 4-laser scanner, with the images generated analysed using the ScanArray Express software. A: 2 μg His-MBP. B: 2 μg His-MBP in the presence of 10 mM maltotriose during incubation and subsequent washing. C: TconTS1-αHel-LD in the absence of maltotriose. D: TconTS1-LD (10 mM maltoriose). E: TconTS2-αHel-LD (10 mM maltoriose). F: TconTS2-LD (10 mM maltoriose). G: TconTS3-αHel-LD (10 mM maltoriose). H: TconTS3-LD (10 mM maltoriose). I: TconTS4-αHel-LD (10 mM maltoriose). J: TconTS4-LD (10 mM maltoriose). K: Array slide before incubation with MBP in the absence of maltotriose. L: Array slide before incubation with TconTS2-αHel-LD in the absence of maltotriose. Inhibited MBP binding to oligomaltose oligosaccharides are cycled in red. Spots cycled in yellow represent internal print controls that are present on all glycan array slides.
(PDF)

S2 Fig Results from glycan array analysis. MBP and TconTS-LD binding to several glycans was determined in the presence (+) or absence (-) of 10 mM mannotriose. No binding was detected to non-listed glycans (see S1 Table for all glycans on arrays).
(PDF)

S3 Fig Amino acid sequence alignment and predicted N-glycosylation sites of TconTS. The primary sequences of TconTS1, TconTS2, TconTS3 and TconTS4 were aligned applying *Geneious Alignment* module of the software Geneious 5.5.5 using the following settings: gap open penalty: 12, Gap extension penalty: 3, Alignment type: Global alignment with free end gaps, Cost Matrix: Blosum62. The predicted *N*-glycosylation sites are illustrated in red triangles and distributed over the catalytic (light blue) and lectin (yellow) domains. No *N*-glycosylation sites have been found in SNAP (orange) and Strep (purple) tags attached to TconTS enzymes.
(PDF)

S4 Fig Binding specificity of TconTS2-LD and enzymatic activity of bacterial and eukaryotic expressed TconTS1. A, B) Binding activities of TconTS2-LD to high-mannose *N*-glycans of immobilised Siglec–2. TconTS2-LD and the antibodies used for detection were consecutively added (triangles) to the immobilised ligands as well as in form of a precomplexed mixture (squares). C) Competitive inhibition of TconTS2-LD specific binding to high-mannose *N*-glycans of Siglec–2. High-mannose *N*-glycans to be used as competitive inhibitor were obtained by EndoH treatment of Siglec–2 (see Methods section for details). αMan: α-methyl-mannopyranoside, Glc: D-glucose. D) Comparison of the specific enzymatic activities of recombinant TconTS1a expressed in *E*.*coli* (bacterial) and CHO-Lec1 (eukaryotic) cells. Velocities shown represent the production of 3‘SL (3‘-sialyllactose) or free Neu5Ac (*N*-acetyl-neuraminic acid) under standard conditions: 100 μg fetuin, 2 mM lactose, 37°C, 30 min.
(PDF)

S1 Table Library of all glycan structures present on our glycan array. (XLS)

S2 Table Sequence similarities of TconTS-LDs. (PDF)

S3 Table Primers for amplification of cDNA fragments encoding TconTS-LDs. (PDF)
