# Correction: Carbohydrate Recognition Specificity of Trans-sialidase Lectin Domain from *Trypanosoma congolense*

**DOI:** 10.1371/journal.pntd.0004344

**Published:** 2015-12-29

**Authors:** Mario Waespy, Thaddeus T. Gbem, Leroy Elenschneider, André-Philippe Jeck, Christopher J. Day, Lauren Hartley-Tassell, Nicolai Bovin, Joe Tiralongo, Thomas Haselhorst, Sørge Kelm

In Fig 2 the glycan name adjacent to ID 1M is listed incorrectly, and in Fig 7, the image in the lower panel is not rotated 90 degrees as indicated. Please see the corrected figures here.

**Fig 2:**
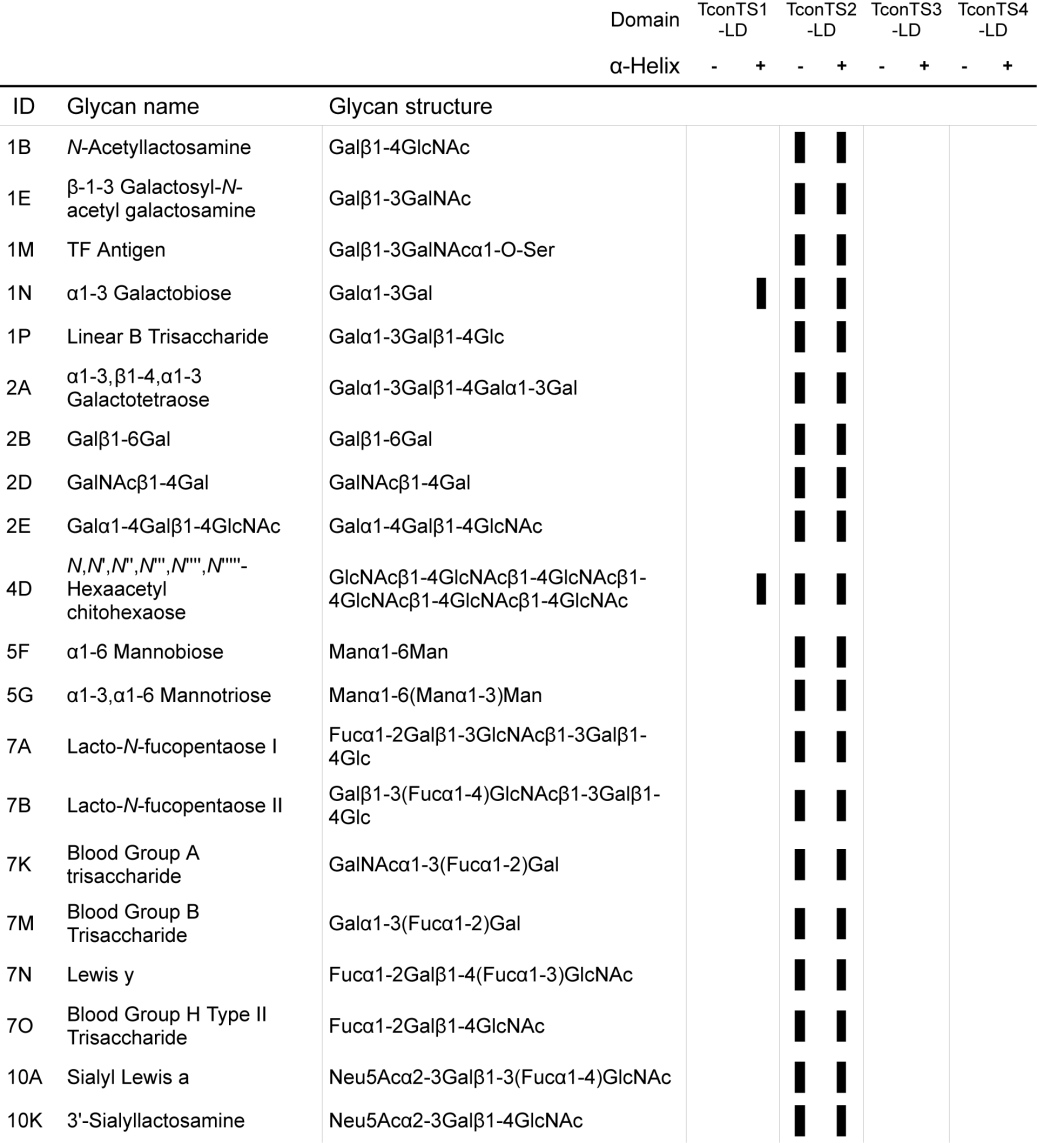
Summary of TconTS-LDs binding to glycans as determined by glycan array analysis. TconTS-LDs binding to the glycan arrays was determined as described under Methods. Black bars indicate glycans bound by the TconTS-LDs. The presence and absence of the α-helix in TconTS-LD constructs is indicated with “+” and “-“, respectively. Further binding data (S2 Fig) and all glycans on the arrays (S1 Table) are available as Supporting Information.

**Fig 7:**
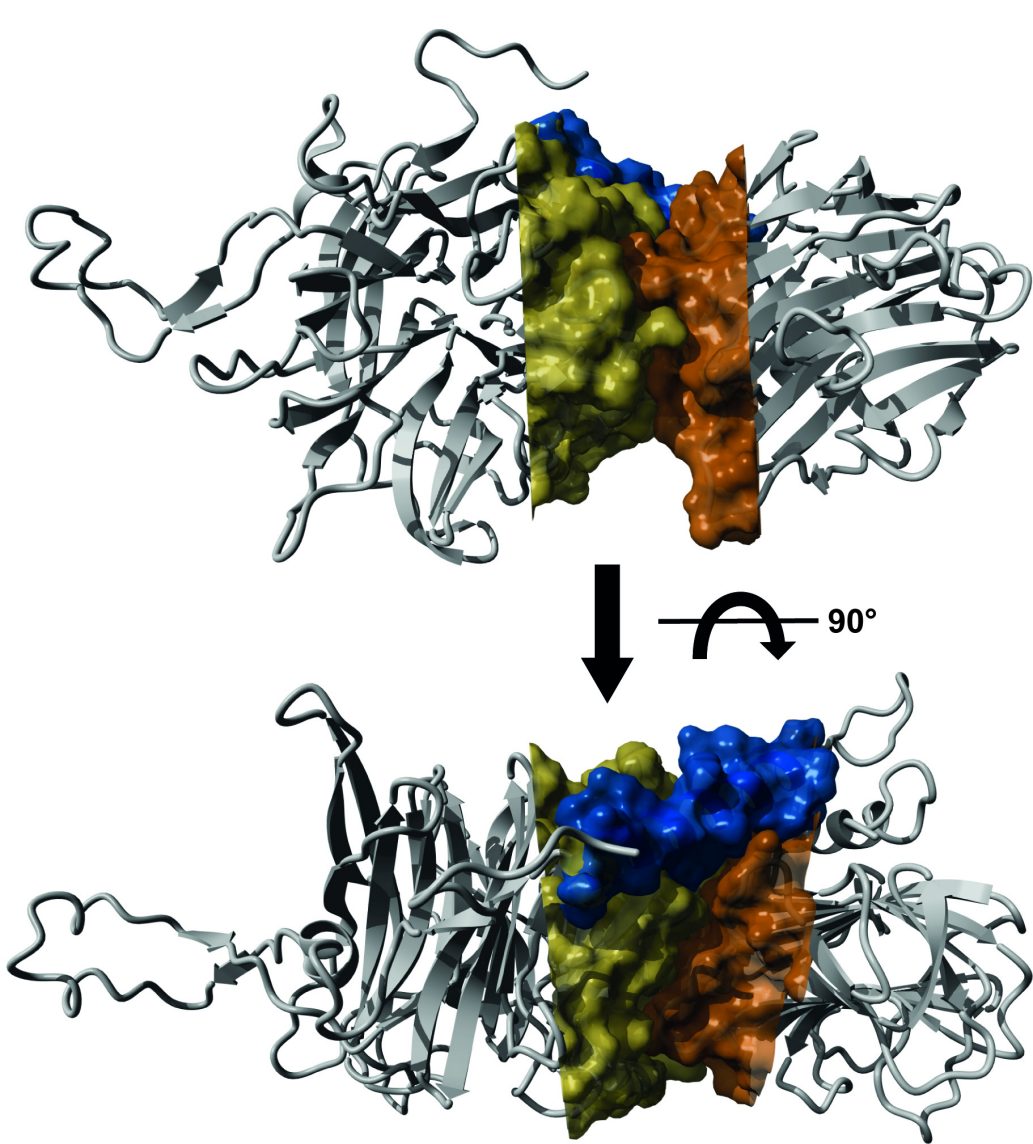
Contact site between TconTS-CD and LD. Homology model of TconTS1 was calculated using the crystal structure of TcTS (PDB: 3b69) as template and the software Yasara. Molecular surface of TconTS1 was calculated using the surface module of Yasara Structure. Illustrated are the parts of TconTS-CD (yellow) and LD (orange), which are in close contact to each other. The α-helix connecting both domains is shown in blue.
