# Supplementary figures and images for: Carbohydrate Recognition Specificity of Trans-sialidase Lectin Domain from Trypanosoma congolense
Source: PLoS Negl Trop Dis. 2015 Oct 16;9(10):e0004120. doi: 10.1371/journal.pntd.0004120 (PMC4608562; doi:10.1371/journal.pntd.0004120)

Figure S1

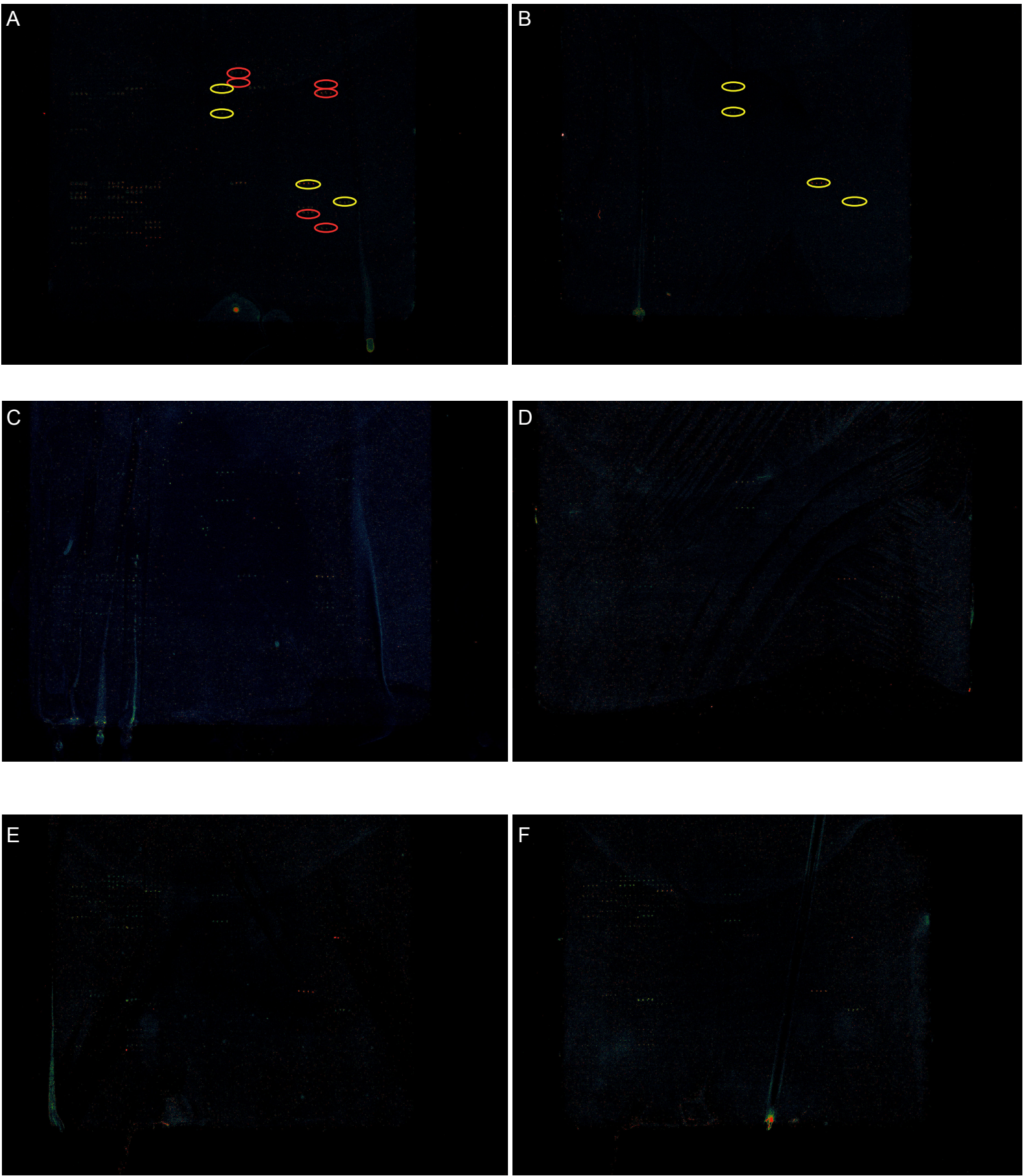

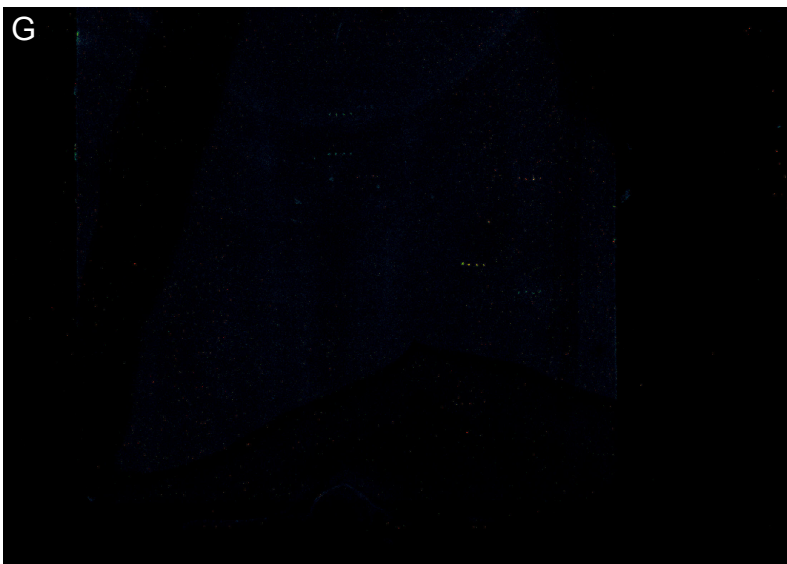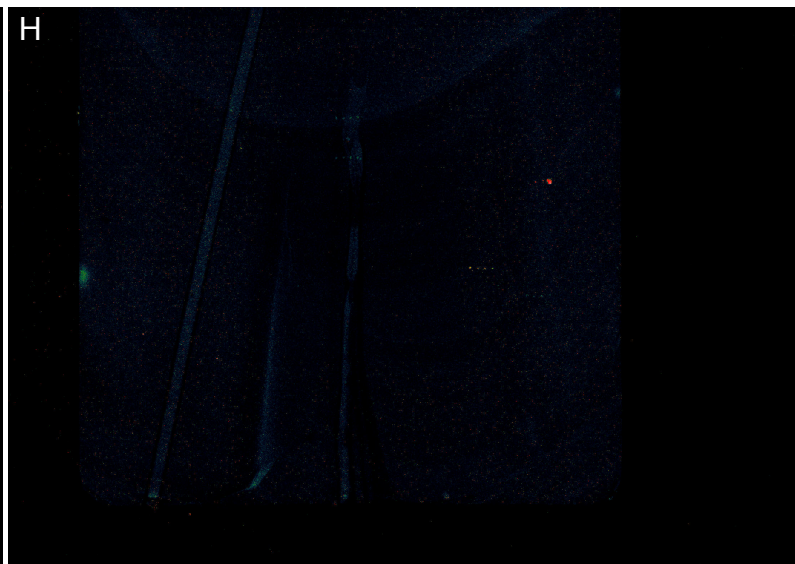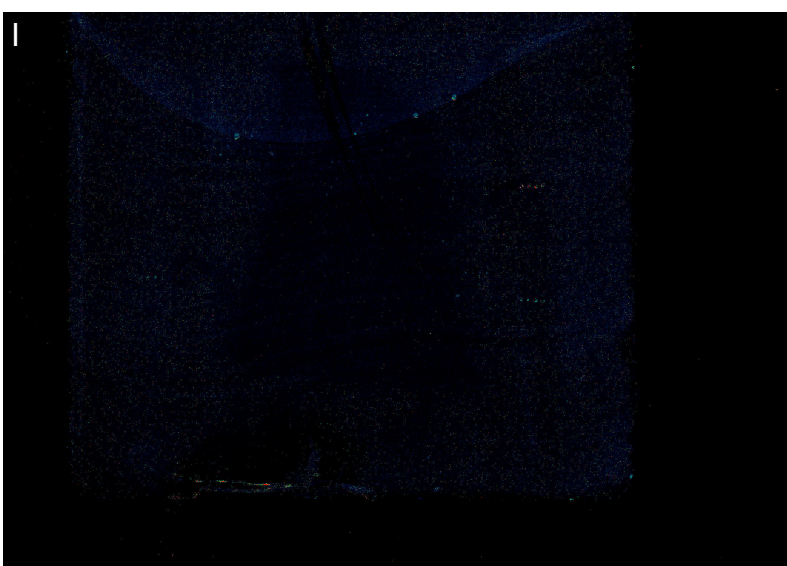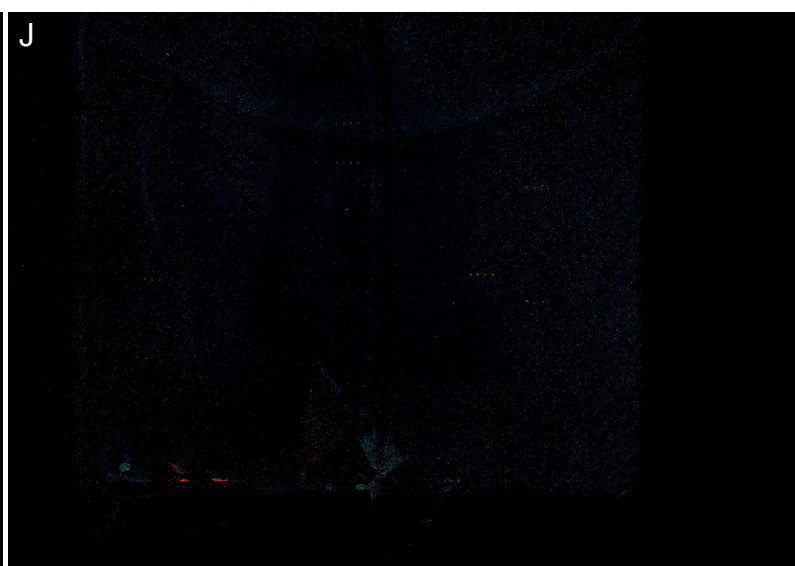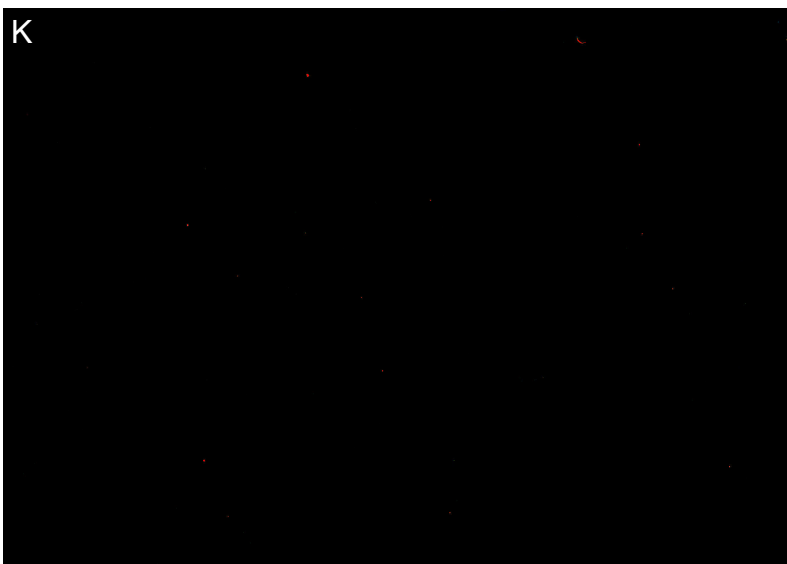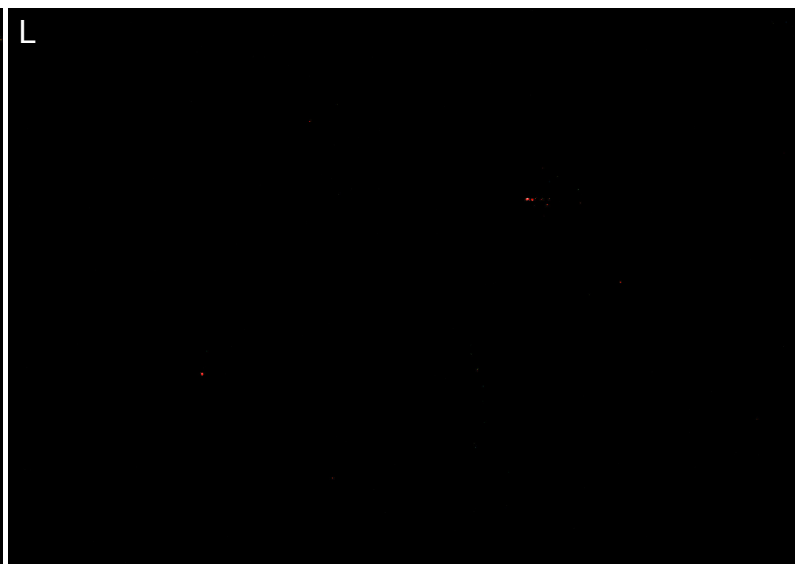

Supplement: S1 Fig — Purified TconTS-LDs (2 μg) were pre-complexed with mouse anti-His, rabbit anti-mouse TexasRed, and donkey anti-rabbit TexasRed and applied to glycan array slides in the presence and absence of 10 mM maltotriose. Following washing and drying array slides were scanned using the ProScanArray Microarray 4-laser scanner, with the images generated analysed using the ScanArray Express software. A: 2 μg His-MBP. B: 2 μg His-MBP in the presence of 10 mM maltotriose during incubation and subsequent washing. C: TconTS1-αHel-LD in the absence of maltotriose. D: TconTS1-LD (10 mM maltoriose). E: TconTS2-αHel-LD (10 mM maltoriose). F: TconTS2-LD (10 mM maltoriose). G: TconTS3-αHel-LD (10 mM maltoriose). H: TconTS3-LD (10 mM maltoriose). I: TconTS4-αHel-LD (10 mM maltoriose). J: TconTS4-LD (10 mM maltoriose). K: Array slide before incubation with MBP in the absence of maltotriose. L: Array slide before incubation with TconTS2-αHel-LD in the absence of maltotriose. Inhibited MBP binding to oligomaltose oligosaccharides are cycled in red. Spots cycled in yellow represent internal print controls that are present on all glycan array slides. (PDF) [file pntd.0004120.s001.pdf]

Fig. S3

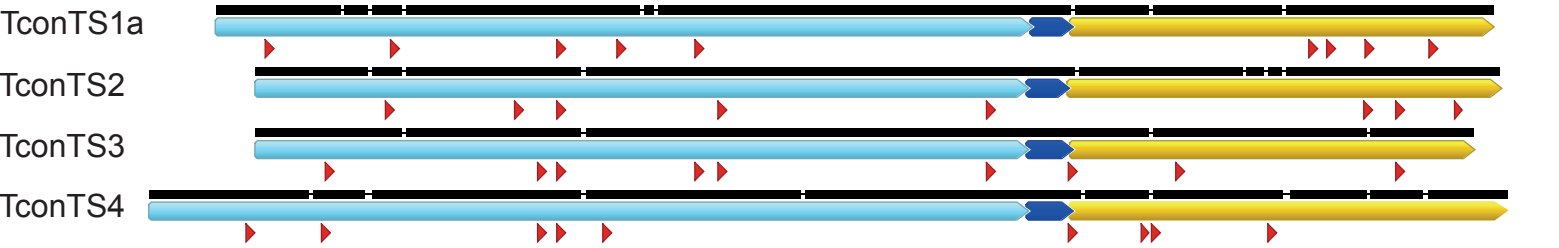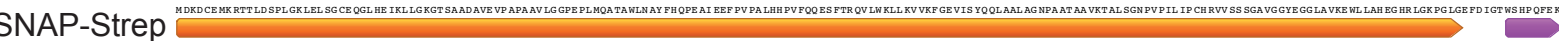

Supplement: S3 Fig — The primary sequences of TconTS1, TconTS2, TconTS3 and TconTS4 were aligned applying Geneious Alignment module of the software Geneious 5.5.5 using the following settings: gap open penalty: 12, Gap extension penalty: 3, Alignment type: Global alignment with free end gaps, Cost Matrix: Blosum62. The predicted N-glycosylation sites are illustrated in red triangles and distributed over the catalytic (light blue) and lectin (yellow) domains. No N-glycosylation sites have been found in SNAP (orange) and Strep (purple) tags attached to TconTS enzymes. (PDF) [file pntd.0004120.s003.pdf]

Fig. S4

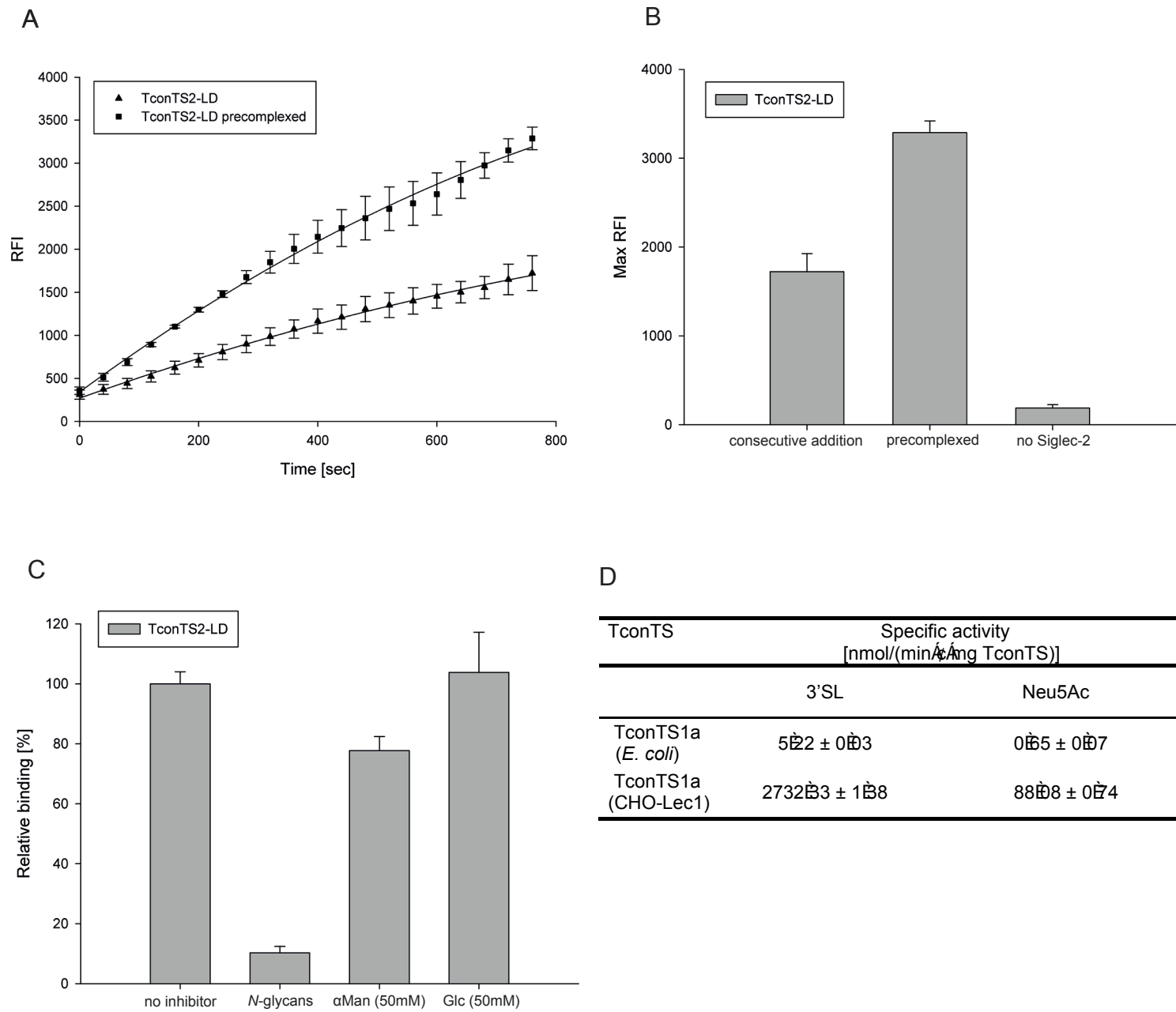

Supplement: S4 Fig — A, B) Binding activities of TconTS2-LD to high-mannose N-glycans of immobilised Siglec–2. TconTS2-LD and the antibodies used for detection were consecutively added (triangles) to the immobilised ligands as well as in form of a precomplexed mixture (squares). C) Competitive inhibition of TconTS2-LD specific binding to high-mannose N-glycans of Siglec–2. High-mannose N-glycans to be used as competitive inhibitor were obtained by EndoH treatment of Siglec–2 (see Methods section for details). αMan: α-methyl-mannopyranoside, Glc: D-glucose. D) Comparison of the specific enzymatic activities of recombinant TconTS1a expressed in E.coli (bacterial) and CHO-Lec1 (eukaryotic) cells. Velocities shown represent the production of 3‘SL (3‘-sialyllactose) or free Neu5Ac (N-acetyl-neuraminic acid) under standard conditions: 100 μg fetuin, 2 mM lactose, 37°C, 30 min. (PDF) [file pntd.0004120.s004.pdf]
