# Supplementary material for: Carbohydrate Recognition Specificity of Trans-sialidase Lectin Domain from Trypanosoma congolense
Source: PLoS Negl Trop Dis. 2015 Oct 16;9(10):e0004120. doi: 10.1371/journal.pntd.0004120 (PMC4608562; doi:10.1371/journal.pntd.0004120)
Supplement: S2 Fig — MBP and TconTS-LD binding to several glycans was determined in the presence (+) or absence (-) of 10 mM mannotriose. No binding was detected to non-listed glycans (see S1 Table for all glycans on arrays). (PDF) [file pntd.0004120.s002.pdf]

Fig. S2

		MBP only		TconTS1-LD			TconTS2-LD		TconTS3-LD		TconTS4-LD	
	α-helix	-	-	-	-	+	-	+	-	+	-	+
	10 mM Maltotriose	-	+	-	+	+	+	+	+	+	+	+
ID	Glycan name											
1B	N-Acetyllactosamine						■	■				
1E	β-1-3 Galactosyl-N-acetyl galactosamine						■	■				
1L	Tn Antigen GalNAcα1-O-Ser	■		■								
1M	Galactosyl-TF Antigen						■	■				
1N	α1-3 Galactobiose					■	■	■				
1P	Linear B-6 Trisaccharide						■	■				
2A	α1-3,β1-4,α1-3 Galactotetraose						■	■				
2B	Galβ1-6Gal						■	■				
2D	GalNAcβ1-4Gal						■	■				
2E	Galα1-4Galβ1-4GlcNAc						■	■				
4D	N,N',N'',N''',N'''',N'''''-Hexaacetyl chitohexaose					■	■	■				
4E	GlcNAcβ1-4MurNAc	■		■								
5F	α1-6-Mannobiose						■	■				
5G	α1-3,α1-6-Mannobiose						■	■				
7A	Lacto-N-fucopentaose I						■	■				
7B	Lacto-N-fucopentaose II						■	■				
7K	Blood Group A trisaccharide						■	■				
7L	Lactodifucotetraose (LDFT)	■		■								
7M	Blood Group B Trisaccharide						■	■				
7N	Lewis y						■	■				
7O	Blood Group H Type II Trisaccharide						■	■				
8A	Sulpho Lewis a						■	■				
8C	Monofucosyl-para-Lacto-N-hexaose IV						■	■				
8E	Difucosyllacto-N-hexaose						■	■				
8G	Lacto-N-fucopentaose VI (LNFP VI)	■		■								
14J	Heparin sulfate	■					■	■				
14L	Chondroitin disaccharide Δdi-OS	■		■								
110	Glcα1-4Glcβ (maltose)	■		■								
240	$(Glc\alpha1\text{-}4)_3\beta$ (maltotriose)	■		■								
241	$(Glc\alpha1\text{-}6)_3\beta$ (Isomaltotriose)	■		■								
8L	Difucosyllacto-N-	■		■								

	neohexaose I										
8M	Difucosyllacto-N-neohexaose II	▮	▮								
8P	Blood Group A Tetrasaccharide	▮	▮								
10A	Sialyl Lewis a	▮	▮			▮	▮				
10C	Sialyllacto-N-tetraose a	▮	▮								
10D	Monosialyl, monofucosyllacto-N neohexose	▮	▮								
10H	Sialyllacto-N-fucopentaose VI (SLNFPVI)	▮	▮								
10K	3'-Sialyllactosamine					▮	▮				
10O	LS-Tetrasaccharide c	▮	▮								
11D	Biantennary 2,6-sialylated-N-glycan-Asn	▮	▮								
12D	Neocarrahexaose-41, 3, 5-tri-O-sulphate (Na+)					▮	▮				
12I	DUA-2S-GlucNS					▮	▮				
12N	DUA-GalNAc-4S	▮	▮								
12O	DUA-GalNAc-6S (Delta Di-6S)					▮	▮				
12P	DUA-GalNAc-4S,6S	▮	▮								
13A	DUA-2S-GalNAc-4S	▮	▮								
13B	DUA-2S-GalNAc-6S (Delta Di-disD)					▮					
13C	DUA-2S-GalNAc-4S-6S	▮	▮		▮	▮	▮				
13F	Hyaluronan fragment (4mer)	▮	▮								
13G	Hyaluronan fragment (8mer)	▮	▮								
13H	Hyaluronan fragment (10mer)	▮	▮								
13I	Hyaluronan fragment (12mer)	▮	▮								
13J	Heparin				▮	▮	▮	▮	▮	▮	▮
13O	HA - 6 10mM	▮	▮								
13P	HA - 8 9.7mM	▮	▮								
14B	HA -12 6.5mM	▮	▮	▮							
14C	HA-14 5.6mM	▮	▮								
14D	HA-16 4.9mM	▮	▮								
390	(Glcα1-4)$_4$β (Maltotetraose)	▮	▮								
391	(Glcα1-6)$_4$β (Isomaltotetraose)	▮	▮								
