# Supplementary material for: Carbohydrate Recognition Specificity of Trans-sialidase Lectin Domain from Trypanosoma congolense
Source: PLoS Negl Trop Dis. 2015 Oct 16;9(10):e0004120. doi: 10.1371/journal.pntd.0004120 (PMC4608562; doi:10.1371/journal.pntd.0004120)
Supplement: S2 Table — (PDF) [file pntd.0004120.s006.pdf]

Table S2: *Sequence similarities between TconTS-LDs*

	**TconTS1-LD**	**TconTS2-LD**	**TconTS3-LD**	**TconTS4-LD**
**TconTS-LD1**	-	42.3% (54.6%)	41.0% (57.7%)	40.7% (51.8%)
**TconTS-LD2**	42.3% (54.6%)	-	39.9% (54.4%)	37.4% (55.1%)
**TconTS-LD3**	41.0% (57.7%)	39.9% (54.4%)	-	45.4% (61.7%)
**TconTS-LD4**	40.7% (51.8%)	37.4% (55.1%)	45.4% (61.7%)	-

The amino acid sequences of TconTS-LD were pairwise aligned using the Blosum62 matrix with a gap opening penalty of 12 and a gap extension penalty of 5. Shown are the percent of positions with identical or similar (giving a positive value in the Blosum62 matrix; in brackets) amino acids.
