# Supplementary material for: Carbohydrate Recognition Specificity of Trans-sialidase Lectin Domain from Trypanosoma congolense
Source: PLoS Negl Trop Dis. 2015 Oct 16;9(10):e0004120. doi: 10.1371/journal.pntd.0004120 (PMC4608562; doi:10.1371/journal.pntd.0004120)
Supplement: S3 Table — (PDF) [file pntd.0004120.s007.pdf]

Table S3. Primers for amplification of cDNA fragments encoding TconTS-LDs

Coding Sequence	Sense primer (5'-3')
His-MBP-TEV	GCCCATGGGCCATCACCATCACCATCAC (*NcoI*)
TconTS1-LD	GC AAG CTT AAC TGC CTC CCG GGC (*HindIII*)
TconTS1-αHel-LD	GC AAG CTT GAC GAG CTG AAA AGC (*HindIII*)
TconTS2-LD	GC AAG CTT TGT CAA CTG AAC AAA AAG CG (*HindIII*)
TconTS2-αHel-LD	GC AAG CTT CTG GAG GAT GAG ATG GAG G (*HindIII*)
TconTS3-LD	GC AAG CTT TGT TCC TCA CCG GAT GGT G (*HindIII*)
TconTS3-αHel-LD	GC AAG CTT CTA GAA GAC GAG CTG GAA AGC(*HindIII*)
TconTS4-LD	GC AAG CTT TGC TCT GCA ACT ACC G (*HindIII*)
TconTS4-αHel-LD	GC AAG CTT CTC GCT GAC GAA CTG AAG (*HindIII*)

Sense primers used in combination with reverse primers (A) GCGTCGACGCCCTGAAAATAAAGATTCTC (*SalI*) for His-MBP-TEV; or (B) GC GCG GCC GCT TAT TTT TCG AAC TGC GG (*NotI*) for the TconTS-LD fragments. The restriction sites used are underlined.
